# Design patterns for wildlife‐related camera trap image analysis

**DOI:** 10.1002/ece3.5767

**Published:** 2019-12-02

**Authors:** Saul Greenberg, Theresa Godin, Jesse Whittington

**Affiliations:** ^1^ Department of Computer Science University of Calgary Calgary AB Canada; ^2^ Freshwater Fisheries Society of BC Research Evaluation & Development Section University of British Columbia Vancouver BC Canada; ^3^ Parks Canada, Banff National Park Banff AB Canada

**Keywords:** camera traps, data encoding and acquisition, design patterns, experience design, human–computer interaction, image inspection, tagging, wildlife monitoring

## Abstract

This paper describes and explains *design patterns* for software that supports how analysts can efficiently inspect and classify camera trap images for wildlife‐related ecological attributes. Broadly speaking, a design pattern identifies a commonly occurring problem and a general reusable design approach to solve that problem. A developer can then use that design approach to create a specific software solution appropriate to the particular situation under consideration. In particular, design patterns for camera trap image analysis by wildlife biologists address solutions to commonly occurring problems they face while inspecting a large number of images and entering ecological data describing image attributes. We developed design patterns for image classification based on our understanding of biologists' needs that we acquired over 8 years during development and application of the freely available Timelapse image analysis system. For each design pattern presented, we describe the problem, a design approach that solves that problem, and a concrete example of how Timelapse addresses the design pattern. Our design patterns offer both general and specific solutions related to: maintaining data consistency, efficiencies in image inspection, methods for navigating between images, efficiencies in data entry including highly repetitious data entry, and sorting and filtering image into sequences, episodes, and subsets. These design patterns can inform the design of other camera trap systems and can help biologists assess how competing software products address their project‐specific needs along with determining an efficient workflow.

## Graphical abstract

We describe design patterns for software that supports how ecologists can efficiently inspect and classify camera trap images for wildlife‐related ecological attributes. Each design pattern addresses a problem and possible solutions to common issues faced by ecologists when inspecting and entering tagging data for a massive number of images. These design patterns inform the design of other camera trap analysis systems, and help biologists assess how competing software products address their project‐specific needs.

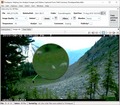

## 1 INTRODUCTION

Camera traps—also known as wildlife, remote, field, or trail cameras—are increasingly used to address a broad range of ecological research and field monitoring applications (e.g., Steenweg et al., 2017; Swann, Kawanishi, & Palmer, 2010). Their basic idea is deceptively simple. First, cameras are located at strategic stations within a geographic study site, where they are positioned to capture activities occurring within a particular field of view (e.g., Tobler, Zúñiga Hartley, Carrillo‐Percastegui, & Powell, 2015). Second, cameras are set up to take images automatically in one of two ways: a *Timelapse* mode where images are taken at regular intervals, or a *motion‐triggering* mode where one or more images are taken whenever movement is detected in the scene. Third, cameras are serviced after a period of time (weeks or months), where field personnel change camera batteries and retrieve the image‐laden SD cards. Fourth, analysts review the set of retrieved images. The analyst examines each image for attributes of interest and encodes those as descriptive or quantitative data. This step is also known as *tagging*. Finally, that data—usually managed and stored in a database or spreadsheet—become the input for the data processing (including statistical analysis) particular to the project.

This paper is primarily concerned with the fourth tagging step described above: how analysts examine images and encode its attributes of interest as data. The number of images typically collected is voluminous: thousands or tens of thousands of images per retrieved camera card easily accumulate to hundreds of thousands and even millions of images per project. Consequently, image examination and data encoding are laborious, time‐intensive, error‐prone, and expensive. It is no wonder that international survey respondents identified image classification as the top challenge in camera trapping (Glover‐Kapfer, Soto‐Navarro, & Wearn, 2019).

Recent research seeks to remedy this burden via automated image recognition, where promising wildlife species detection and identification rates have been reported (e.g., Norouzzadeha et al., 2018; Schneider, Taylor, & Kramer, 2018; Tabak et al., 2018; Yousif, Yuan, Kays, & He, 2019). Unfortunately, image recognition for camera traps is still in its formative stage. It is limited in what can be recognized. For example, somewhat easy to extremely difficult recognition challenges range from: detecting if wildlife is present, identifying the species, identifying individuals, determining animal health, to distinguishing animal behaviors. Image recognition also: requires a model trained on domain‐specific images; incurs varying rates of classification errors (false positives and false negatives); performs less well with new camera placements (due to different backgrounds); and is currently poorly integrated in the analyst's software and workflow. Even if it was available, analysts would still have to verify recognition predictions and correct erroneous ones. Manual methods will likely dominate for years to come, especially when additional attributes beyond simple species detection are required (but see Section 8 below).

In the past, analysts resorted to off‐the‐shelf generic software when tagging images, such as a stock image viewer to view images, and a separate spreadsheet package to record data. More recently, researchers and corporations have developed specialized software packages to support camera trap analysis (e.g., Bubnicki, Churski, & Kuijper, 2016; Ivan & Newkirk, 2016; Krishnappa & Turner, 2014; Reconyx Inc, 2016; Scotson et al., 2017; Swanson et al., 2015; WildTrax, 2019; Young, Rode‐Margono, & Amin, 2018). Such camera trap software systems should, of course, include user interface features that encourage efficient human inspection of images and encoding of its data attributes. Those interface features should be based on a firm understanding of what analysts do when examining and encoding images, including mitigating human performance bottlenecks. However, most descriptions of these systems often provide only sparse details and discussions (if any) of the problems faced by analysts that the system purportedly solves, the benefits of the particular interface features provided, and how the particular solution offered can be generalized to other systems. Thus, there is a gap in how the lessons learnt from developing such systems could be applied to evaluate current software and/or developing next generation camera trap software interfaces. As a recent extensive World Wildlife Fund report on best practices for camera‐trapping summarizes:

Importantly, no single software package has emerged as a favourite amongst camera trappers, and lots of very different solutions to the problem of camera trap data management are currently being trialled… For any given camera trap project…, this makes it difficult to decide which software package to commit to. Many large camera trap projects … have ended up designing their own systems from scratch. (p. 146)

The various software options available differ greatly in their approaches …and you may need to test various options before deciding which one satisfies your requirements and most efficiently fits into your workflow (p. 150). (Wearn and Glover‐Kapfer, 2017)

Our goal in this paper is to describe and explain *user interface design patterns* for software supporting how wildlife biologists perform camera trap image analysis during the tagging step. By way of background, the notion of design patterns was first introduced by architect Christopher Alexander as a documented reusable and proven solution to commonly occurring architectural design problems (Alexander, 1977). Design patterns are typically derived by examining existing solutions to design problems (which may include “folk” solutions) and generalizing them. Design patterns were later advocated as a way of describing common solutions to typical software engineering problems (Gamma, Helm, Johnson, & Vlissides, 1994) and to human–computer interaction problems (Borchers, 2001). Design patterns are much more than a feature list, for that they provide the rationale behind a feature in a general and reusable manner. Design patterns are usually structured as a name, a problem that explains it, and a design approach to a solution that solves the problem. Importantly, a design pattern is not a finished design. Rather, it is a description or template for how to solve a problem that can be used in many different situations.

We base our design patterns on both our understanding of what biologists require and the user interface features that support their needs from over 8 years of developing and deploying our freely available Timelapse Image Analysis system^1^ (Greenberg, 2019; Greenberg & Godin, 2015, 2). For each design pattern presented, we describe the problem faced by the image analyst, a design approach that solves that problem, and a concrete example of how Timelapse addresses that design pattern.^3^

While this paper concerns the design of software, we stress that it is highly relevant to wildlife biologists. It is the biologist—not programmers—that should determine and decide upon what camera trap design features are important to their work. We also recommend that biologists should be part of any camera trap software design team, where they should be the ones motivating what requirements should be included, how requirements should appear in the software, and how software features should be considered in the workflow.

## 2 THE DIVERSITY OF CAMERA TRAP RESEARCH PROJECTS, GOALS, AND IMAGES

**Figure 1:**
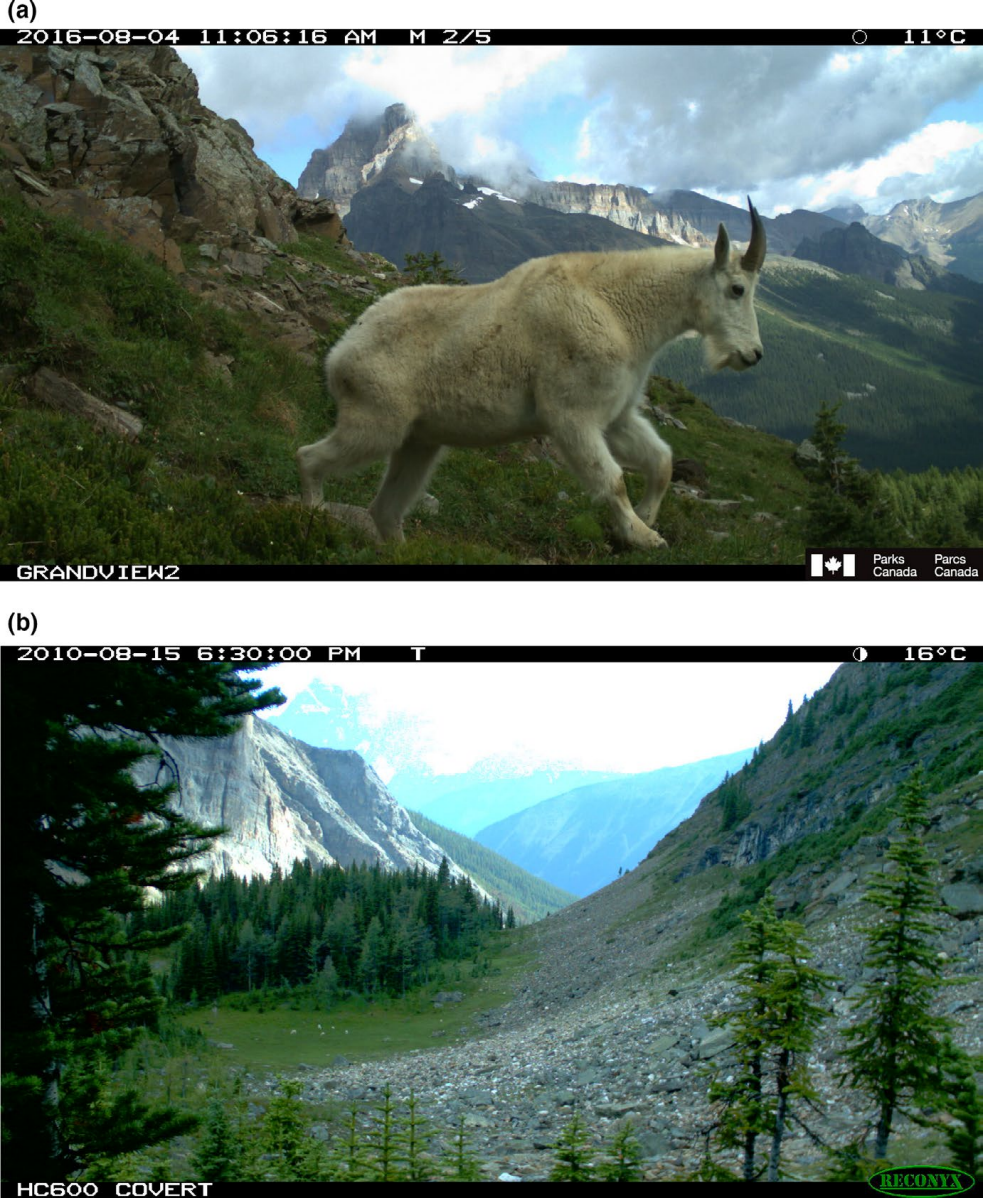
The diversity of images produced by two camera traps. (a) A close‐up shot of a mountain goat. (b) Mountain goats are barely visible in the meadow as white dots.

Camera traps are used to address a wide diversity of ecological research and management objectives and associated taxa. This diversity leads to large differences in how cameras are situated and configured, the kinds of images collected, how analysts would examine those images, the attribute data recorded from images, and how the collected data would be subsequently analyzed. For example, Figure 1 illustrates differences between two images of the same species with varying research objectives. In Figure 1a, the camera was set to motion‐triggering to capture close‐up views of mountain goats (*Oreamnos americanus*) as they passed by. Attributes of the goat could then be analyzed (e.g., species identification, sex, estimated age, individual identification, etc.). In Figure 1b, the camera was set to Timelapse mode that took an image every 5 min in order to monitor the presence and activity of a herd of goats over time on a distant pasture and mountain‐side. Attributes of herd activity could then be analyzed (e.g., counts, duration in the meadow, ratio of kids to adults, etc.). Figure 1b includes a small herd in the green pasture, each goat just visible as white dots.

Perhaps, the most familiar uses of camera traps and the subsequence analysis of its images are in some form of in situ wildlife monitoring (e.g., Burton et al., 2015; Steenweg et al., 2017). For example, in a review of 266 camera trap publications over a 6‐year period, Burton et al. counted a range of ecological objectives and responses metrics including: relative abundance (43.6%), presence–absence (41.4%), behavior such as activity patterns and diet (32.3%), population density (15.8%), and occupancy (15.4%) (Burton et al., 2015, p. 678). Other examples illustrate further diversity of objectives:

- monitor species diversity and inventories (e.g., Ahumada, Hurtado, & Lizcano, 2013; Glover‐Kapfer et al., 2019; O'Brien & Kinnaird, 2010),
- measure population abundance, density, and distribution of marked and unmarked populations (e.g., Goswami, Madhusudan, & Ullas Karanth, 2007; Heilbrun, Silvy, Peterson, & Tewes, 2006; Karanth & Nichols, 1998; Rowcliffe & Carbone, 2008; Royle, Fuller, & Sutherland, 2018; Whittington, Low, & Hunt, 2019; Whittington, Hebblewhite, & Chandler, 2018)
- examine multi‐species dynamics (Swanson et al., 2015),
- estimate population trends (e.g., Karanth, Nichols, Samba Kumar, & Hines, 2006),
- correlating wildlife abundances to anthropological stressors (e.g., Fisher & Burton, 2018), including human activity, and to mitigation efforts (e.g., Pollock, Nielsen, & St. Clair, 2017; Whittington et al., 2019),
- quantify animal behavior and success rates in DNA hair capture sites (Clevenger & Sawaya, 2010),
- linking seasonal plant phenology to climate change and wildlife distributions (e.g., Laskin et al., 2019; Mills et al., 2018), and
- quantifying how seasonal changes in coat color are influenced by climate change (e.g., Mills et al., 2018).

Camera traps can also help answer very specific research questions. For example, García‐Salgado et al. (2015) analyzed the diet of nesting raptors by examining images for prey deliveries to nests. Rollack, Wiebe, Stoffel, and Houston (2013) similarly deployed cameras around nests, but in this case to study the breeding behaviors of turkey vultures. Jumeau, Petrod, and Handrich (2017) used camera traps to estimate the effectiveness of wildlife crossing structures for small mammals.

Camera traps are also used to monitor and analyze human activity. Examples include: counting the number of anglers actively fishing in order to estimate angling effort in small lakes fisheries (Greenberg & Godin, 2015); evaluating the influence of human disturbance on wildlife (e.g., Blake, Mosquera, Loiselle, Romo, & Swing, 2017; Oberoslerab, Groff, Lemma, Pedrini, & Rovero, 2017), quantifying levels of human use and type of human activities (e.g., Campbell, 2010; Fairfax, MacKenzie Dowling, & Neldner, 2014); and detecting types and occurrences of illegal activity in wildlife sanctuaries including identifying perpetrators (e.g., Hossain et al., 2016).

The above is just a very small sampling to indicate the diverse use of camera traps (e.g., see Section 5 in Steenweg et al., 2017; Wearn & Glover‐Kapfer, 2017). While there may be some overlap in the kinds of images gathered across projects, we can easily expect differences between projects in: the kinds of images that are captured, the data attributes that would be encoded from these images, and the ways analysts would have to examine those images to extract and record that data. Our design patterns reflect this diversity of project objectives. Some design patterns are broadly applicable and useful in all kinds of image classification, while others may be of use in narrower suites of projects. More generally, software based on these design patterns will include tools for examining images, will provide a flexible user interface for efficiently encoding project‐specific attributes, and will simplify data entry by automatically extracting available image features as data. Collectively, it can substantially increase project efficiency, increase data quality and improve the user experience.

## 3 METHODOLOGY: DECONSTRUCTING THE DESIGN OF TIMELAPSE INTO DESIGN PATTERNS

Our methodology for understanding how analysts classify data and what user interface design patterns would be useful was derived from deconstructing our real‐world experiences designing, implementing, and refining the Timelapse open software system. Timelapse was specifically developed to help analysts inspect and classify camera trap images via tagging. Timelapse is conceptually simple: it displays images to analysts, along with a variety of project‐specific (and user generated) attribute fields that they can efficiently fill in to describe the image. Yet its design goes far beyond that, for it, includes many features addressing the subtleties of the analyst's workflow.

Timelapse evolved through many versions over its 8 years. Its capabilities were designed to meet a broad variety of ecological needs as requested from a diverse international user community comprising different agencies (government, industry, university, and independents) and biologists (researchers, practitioners, and students). Most had differing projects and goals (e.g., wildlife monitoring, angling effort in fisheries, environmental monitoring, human monitoring, etc.). Our requirements, analysis, and various redesigns of Timelapse were further informed by the following.

- We had ongoing discussions with both project managers and analysts about their camera trap image analysis needs and existing workflow.
- We held observational studies of analysts using Timelapse as they did their work (e.g., Greenberg & Godin, 2015), using standard techniques in the field of human–computer interaction (e.g., Shneiderman et al., 2016). We observed and interviewed the technicians analyzing images, paying particular attention to their workflow, problems encountered, and bottlenecks;
- We collected feedback from analysts who had used Timelapse to inspect millions of images (e.g., problems, feature requests, bottlenecks).

We emphasize that our methodology followed an iterative versus one‐time design process. We began with understanding the requirements of a single small‐lakes fisheries agency (Greenberg & Godin, 2015). We repeated our methodology as other agencies from different domains and with different project needs came on board. Our ongoing discussions, observations, and feedback with those agencies helped us understand and articulate the subtleties and variations in the workflow that arose due to various factors. For example, the type of images captured at particular sites differed considerably, which often led to workflow alterations in how technicians analyzed images. As well, particular subprojects required analysts to examine and encode different image features as data, again resulting in workflow differences. We used this knowledge to update the Timelapse design, albeit with the constraint that it had to remain a tool usable by its broad community. That is, new interface features would be added only if they were potentially valuable to a broad range of projects and users, or—at the very least—could be ignored if they were not needed.

The remainder of this paper deconstructs and generalizes as design patterns the key workflow tasks and problems seen, and how they informed the corresponding Timelapse interface design solutions. Each section is organized around an issue that relates particular problems faced by analysts. Each problem is followed by a named design pattern. This includes a general description of an interface design solution that mitigates that problem and a concrete example of how Timelapse realized that design pattern. We also invite the reader to run Timelapse as they read the design patterns, as this can help them better understand the nuances of the proposed solution.

While we highlight how Timelapse instantiates a particular design pattern solution, we stress that the design pattern is more general than that, as it can also inform alternate software designs. For example, designers can selectively incorporate particular design solutions as seen in Timelapse into their own systems. Alternately, designers can use our design pattern problem descriptions and general solutions to create their own novel, alternate ways to solve those problems. Finally, project managers can match design patterns against their project needs, and then evaluate competing software solutions to see whether those solutions have interface features that support the design patterns relevant to their project.

We recognize that other camera trap software systems may offer similar or alternate solutions to our design patterns. However, we restrict our example solutions to Timelapse, as a comparative review and analysis are beyond the scope of this paper. Still, the design patterns supplied here should allow readers to reconsider whether and how other software solutions address particular design problems and may even help them identify problems and design patterns that are outside of what we provide below.

## 4 ISSUE: DATA CONSISTENCY

The ultimate goal of the analyst is to enter attribute data that reflect the contents of the images. Statistical analysis of attribute data collected from those images typically occurs later as a separate step. The value of attribute data depends upon its consistency (explained below) and quality. Thus, data entry protocols for what attributes to collect must be developed prior to image classification.

### 4.1 Problem—The data required and how they are named may be inconsistent between analysts

Projects typically involve multiple cameras located at multiple stations at one or more study sites. In turn, this can generate a large number of distinct *image sets*,^4^ each containing many thousands of images. Multiple analysts may be involved (perhaps including volunteer citizen scientists), each analyzing the images within a particular image set. A key issue is maintaining data consistency across different image sets and different analysts, that is, where all analysts are inputting consistently formatted data into a set of consistently named data fields. Without data consistency (e.g., if each analyst idiosyncratically specified what data should be encoded from images, in what format, and under what name), it would be extremely difficult to make sense of the data across analysts and image sets.

#### 4.1.1 Design pattern: Specify and deploy a common data schema

The project manager should initially decide what data should be collected from the image sets and communicate those needs to the analysts as a standardized computer‐readable *schema* that defines and specifies the data of interest. Analysis of image sets by all analysts should then be based on that schema. The image analysis software should enforce the schema, where deviations from that schema are discouraged unless absolutely necessary. The schema should define the required data fields, how they are named, their data type, their format, and constraints on what values they allow.

#### 4.1.2 Timelapse example

**Figure 2:**
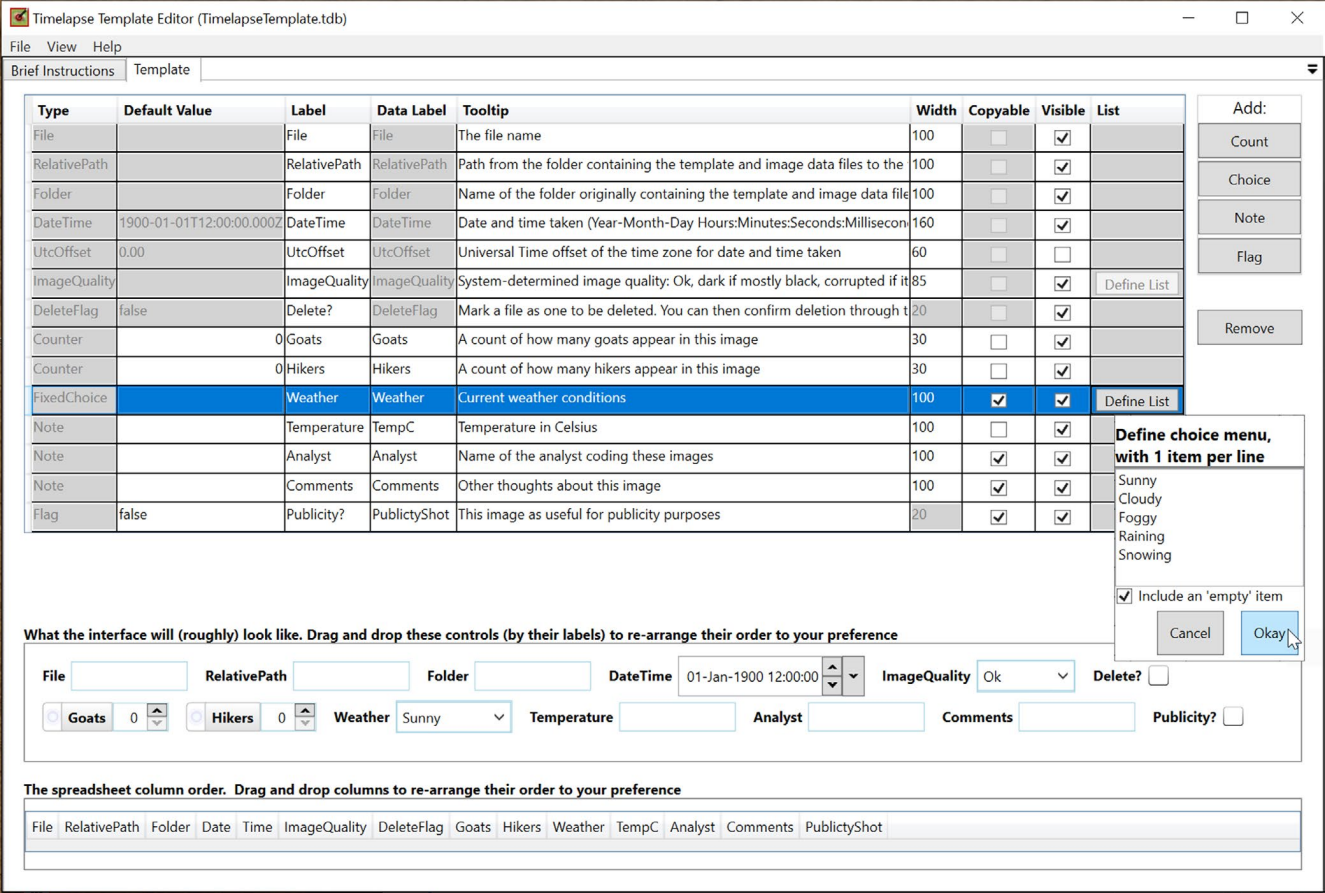
The Template Editor. The project manager defines the attribute data of interest (the schema) as well as the associated user interface specifications by form‐filling (top pane). The middle pane generates a preview of the user interface data entry controls that will be seen by the analyst. The bottom pane shows how the data of interest will be stored as columns within a database table. Interface controls and spreadsheet columns can be re‐ordered by dragging them to the desired location

**Figure 3:**
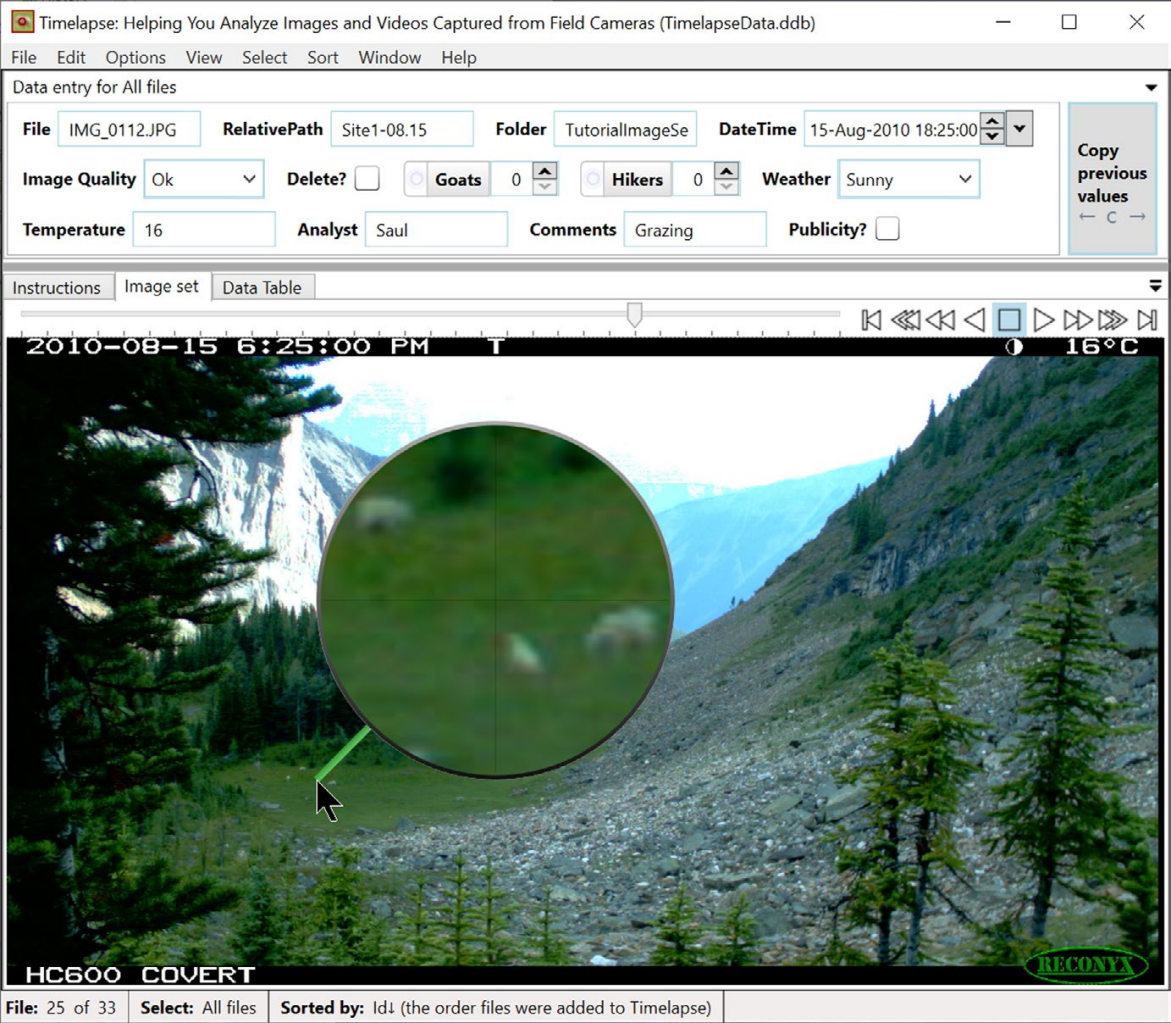
Timelapse Image Analyzer. The analyst uses Timelapse to navigate and inspect images (lower panel) and to enter data describing an image's features of interest (top panel). The data entry controls shown in the top panel were automatcially constructed from the schema information specified by the project manager through the template editor illustrated in Figure 1

Project managers use a Timelapse utility called the *template editor* (Figure 2) to construct a template file that defines the data schema. Analysts place the data template file in the folder containing the image set. When the actual Timelapse application is opened (Figure 3), it uses the template schema to build the user interface and to construct the database table that will ultimately contain the data entered by the analyst. This enforces the data schema.

Figure 2 illustrates a screenshot of the template editor in action. In this example, the project manager has specified a fairly simple schema used to count how many goats and hikers are seemed and to track environmental conditions and a few other attributes.^5^ Generally, each row in the form specifies all attributes of a particular data field, while each column names the attribute. The template editor allows project managers to easily create project‐specific schemas. Each attribute field of the schema is developed with the following options:

- *Type *indicates the data type and its format. For example, the type *DateTime* and *UTCOffset* follow the international standard for specifying dates. *Counters* are positive integers and are usually used for counting entities in an image. *Flags* can only contain true/false values. *Notes* are free‐form text fields. *Choices* are constrained to a limited number of possible values provided by the project manager. For example, a field labeled “Weather” could be constrained to the values “Sunny,” “Cloudy,” “Foggy” etc., while a field named “Species” could list—and eventually allow an analyst to select from—all possible species of interest in that ecology.
- *Data Label *names the data field containing that data, that is, the column name as it would appear in a database table or a spreadsheet.
- *Default Value* indicates the initial value of that data, which will be applied to every image seen by Timelapse.
- *List *defines the allowable entries for Choice data types. Selecting “Define list” raises a small window where the manager can type in those entries (see Figure 2, lower right side).

### 4.2 Problem—Data schema terminology may not be in the analyst's language

Computer systems often ask users to enter data by either filling in rows in a table, or via data entry widgets (textboxes for text entry, menus for selecting choices, etc.). These are often labeled in some manner (e.g., column names in a table or a name associated with the data entry widget). Labels are important, as analysts need to understand what data they are expected to enter. The issue is that the terminology used may not be in the analyst's language. For example, systems may use the database field name to label a data entry widget, but these names may not be understandable (e.g., acronyms, abbreviations, technical terms, ambiguous meanings, etc.) or insufficient to describe what the analyst should enter. This can lead to inconsistencies between the project manager's expectations of the data required versus the analysts' interpretation of what data should be entered. A related issue is that the schema may include fields that are of little interest to the analyzer, such as fields they are not expected to review or fill in. Their inclusion in the analyst's user interface would add clutter and perhaps confusion, or have the analyst fill in fields unnecessarily. Finally, because the project manager and all analysts need to communicate to one another, the terminology used to identify that data should be common. These terminology problems are particularly endemic to analysts who inspect images only occasionally, as they may forget what particular terms mean, and to citizen scientists with minimal training.

#### 4.2.1 Design pattern: Present the data schema in the analyst's language

The data entry interface presented to the analyst should be expressed clearly in familiar words, phrases, concepts, and explanations rather than in system‐oriented terms. Nonrelevant data items should be removed (Nielsen, 1993, Chapter 5). One way to do this is to associate the terms in the data schema with a more understandable terminology and descriptions of what data they are expected to enter, and whether or not particular data items should be displayed to the analyst. While the interface seen by the analyst would be constructed using that terminology, the data entered by the analyst would be stored under the data labels provided by the data schema, thereby maintaining data consistency.

#### 4.2.2 Timelapse example

Using the Timelapse *Template Editor*, the project manager specifies the terminology of data entry controls, that is, the interface controls associated with every data field used by the analyst to enter data. For example, consider the data field with the data label of “GoatCnt” in Figure 2. In the *Label* column, the manager specified “Goats” as a more human‐readable alternative to that data field. In the *Tooltip* column, the manager provided a brief help explanation of what should be entered: “A count of how many goats appear in this image.” By unchecking the “Visible” checkbox, the manager has indicated that the “UtcOffset” data field should not be displayed to the analyst. As the manager performs these actions, a live preview of the user interface (Figure 2, middle pane) reflects the actual user interface that will be seen by the analyst when using the Timelapse system (Figure 3, top pane). Both illustrate how the data controls adopt the analyst‐oriented terminology and explanations specified in the template (e.g., the “Goats” control and the displayed tooltip). When the analyst enters data in that control (Figure 3), it is stored in the corresponding data field (e.g., “GoatCnt”).

### 4.3 Issue: Data input errors are commonplace

When analysts enter data, they may inadvertently introduce errors into the stored data. For example, the entered data may be outside of what is expected (e.g., nonnumeric characters entered into an integer data field, Yes/No instead of True/False as expected by a boolean data field). As another example, the entered data may not be in the correct format (e.g., date may be incorrectly entered as “mm/dd/yyyy” order instead of the expected “dd//mm/yyyy”).

#### 4.3.1 Design pattern: Data entry controls should minimize input errors by constraining input to the data field's type

Input controls should provide visual hints of what input is accepted, and should only accept input that matches the data field's type and format. This pattern is now common in many modern user interfaces, where myriads of input controls suggest and constrain what can be entered into them.

#### 4.3.2 Timelapse example

Timelapse generates its user interface from the description provided in the template editor (Figure 2), where each data entry control is based on its corresponding template specification (Figure 3, top pane). The data entry control constrains the input, where only valid data can be entered into it. For example, an analyst interacting with the DateTime data entry control (Figure 3, top pane, right side) can only enter or edit valid date and time values. Counter controls (e.g., Goats, Hikers) only accept positive integers (either by typing or by mouse interaction). Choice data controls (e.g., Image Quality, Weather) restrict the analyst to selecting from a pop‐up menu containing only valid text entries. Flags (e.g., Delete?, Publicity?) present themselves as a checkbox, where the control itself translates its “checked” state to the two allowable data storage values of “true” or “false.” In all cases, the analyst's data entry is constrained by the corresponding data control to allow only legal values, which reduces possible input errors significantly.

## 5 ISSUE: INSPECTING IMAGE FEATURES

A major part of the analyst's tasks is to inspect the image to discover (and record as data) features of interest. Yet inspection can be problematic for some images, especially when the features of interest are not discernable at a glance.

### 5.1 Problem—Relevant image details may be difficult to examine

**Figure 4:**
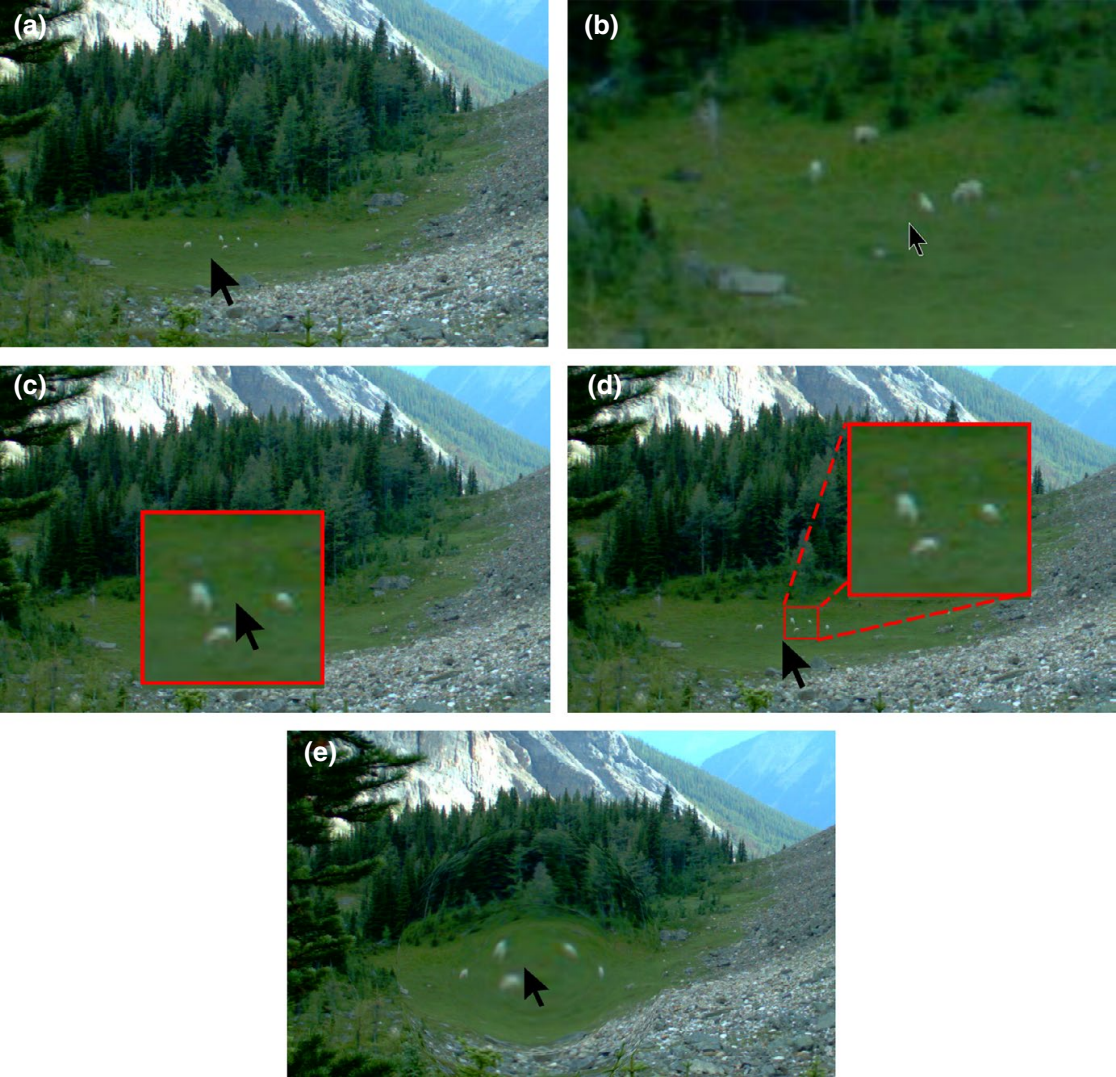
Various approaches to examining details via magnification. (a) The unaltered view. (b) Pan and zoom. (c) Standard Magnifying lens. (d) Offset magnifying. (e) Fisheye lens.

Depending on what is captured in the image's field of view, analysts may have to inspect small image details in order to classify what is there. One example is camera images that capture large fields of view, where the items of interest are very small. Figures 1b, 3 and 4a illustrate such an image, where the camera is oriented to capture distant goats as they wander through a mountain‐side and meadow. Other examples include cases where the analyst has to identify small animals, or details of that animal (e.g., sex, health), or where the image only displays a portion of that animal (e.g., because of occlusion by vegetation, or because the animal is only partly in the camera's field of view).

#### 5.1.1 Design pattern: Allow the analyst to examine image details through magnification

Image magnification can help the analyst examine small image features. However, due to the number of images inspected, the analyst's interaction with the provided magnification technique must be efficient to use. Various magnification interaction techniques are known. The most common is perhaps a *pan and zoom* facility (Figure 4b), which allows the analyst to zoom (magnify) a particular image region (called the *focus*). While powerful, zooming into local detail incurs the cost of losing *global context*, that is, only the zoomed in portion of the image is visible, which means informative details outside of the focus cannot be seen at the same time.

Another common approach mimics a *magnifying lens* (see Figure 4c), where the area under the cursor (the focus point) is overlaid by a small zoomed in region. The area being magnified is immediately updated as the cursor is moved. Again, there is a trade‐off: while the magnified areas show the focus area under the cursor, it occludes some of the surrounding global context as it overlays it. For example, Figure 4c magnifies 3 of the goats, but at the cost of occluding the other two nearby goats.

Far more sophisticated magnification approaches have been developed in the field of *information visualization* (e.g., Shneiderman et al., 2016; Spence, 2014 Ch. 12). For example, an *offset magnifying lens* avoids occlusion by offsetting the magnified area away from the cursor's focus area (Ware & Lewis, 1995). As Figure 4d illustrates, the small square by the cursor is the area to magnify, where that zoomed in area is shown offset in the larger square. Thus, the analyst can simultaneously see both the unzoomed and zoomed in area at the same time. Another approach is *focus plus context magnification*. For example, a *fisheye lens* distorts an image to provide magnification in place (Carpendale, Light, & Pattison, 2004). As Figure 4e illustrates, the highest magnification is focused under the cursor, where a drop‐off function applies progressively less magnification away from the cursor. The advantage, as seen in Figure 4e, is that local detail is shown in place within the global context. This also avoids separation of the magnified versus unmagnified image as evident in Figure 4b,c.

Of course, the value of magnification is affected by image fidelity. Some systems may, for example, reduce an image's display resolution for performance purposes with the side effect of comprising image details. The fewer the pixels read in, the less memory required and the faster images can be displayed. The trade‐off is that magnification would then produce “fat pixels” (*aka* blurry images) rather than details. Fortunately, various image processing techniques are known that can efficiently display the whole image at low fidelity, while reading in high resolution details for only the magnified regions (Carpendale et al., 2004).

#### 5.1.2 Timelapse example

Timelapse contains several methods for rapidly examining image details through magnification.

1. **Zoom and pan.** The analyst can zoom and pan into any part of the image using the scroll wheel and the mouse. Zooming occurs at the cursor location, while panning to a particular image region is done by dragging the image with the mouse. Timelapse's zooming and panning features also include nuances that support how an analyst would use it over multiple images. First, the analyst can “bookmark” a particular zoomed in area, where she can flip between the zoomed and normal image with a single keypress, thus maintaining some sense of how the zoomed‐in focus relates to the global context. Bookmarks have other advantages. For example, if the analyst was interested in animal activity in the pasture seen in Figure 3, she could zoom into that pasture and bookmark it. When checking other images for activity in that pasture, she can use that bookmark to zoom into the same corresponding area. Second, zoom/pan levels are maintained when navigating between images. For example, the analyst could zoom into the pasture of Figure 3 and then see how the goats have moved around that pasture by navigating to the next few images.
2. **Offset magnifying lens.** Similar to Figure 4d but with different visuals, the analyst can turn on a magnifier that displays zoomed‐in image of the area around the cursor: the lens is offset to avoid occluding that area. Figure 3 illustrates the Timelapse magnifier in action, where the analyst is using it to detect and examine a herd of goats. The analyzer can easily scan the image details for features of interest by dragging the magnifier, whose magnified content is instantly updated. The analyst can also quickly adjust the amount of magnification through a few keystrokes.

### 5.2 Problem—The presence of small entities may be difficult to notice

Various projects use cameras in Timelapse mode, where periodically taken images capture a very wide field of view. We already saw how Figures 1b and 3 illustrate one actual example, where the camera was located to capture distant goat activity in a pasture and on a mountain side. Another real example includes cameras positioned to capture distant anglers on and around a large lake area (Greenberg & Godin, 2015). The issue is that analysts may not notice the presence of these small entities. This becomes more problematic when a run of images being examined have nothing in them, as analysts expect that pattern to continue. Magnification, while somewhat helpful, is best used to examine details *after* an entity has been noticed.

#### 5.2.1 Design pattern: Enhance the noticeability of small entities within images

Various techniques can enhance how the analyst can notice small entities in a scene by making them visually distinctive.

*Animation of image sequences* visually highlights changes that occur by rapidly switching between images. Because the background scene is reasonably constant, the appearance, disappearance, and movements of entities within the scene are often very noticeable.

*Image processing through image differencing* compares, pixel by pixel, the current image against the previous and/or next image. A new image is generated from that comparison, where (for example) a white pixel is drawn if the compared pixels differ significantly in brightness and color (set by threshold values), and black otherwise. The resulting image visually highlights the differences in white, while removing the somewhat static background. Because entities appear, disappear, and move around a scene, the differenced image will display that entity as a white blob (usually in the shape of the desired entity) against a black background. Other image processing techniques may also help, such as motion tracking that track the position of an object over subsequent frames. We note that the effectiveness of image processing techniques can be compromised when large visual differences occur between the images, such as dramatic changes in image lighting, motion of nearby grass and branches affected by the wind, and even slight changes of a camera's position (e.g., due to wind effects on the tree, it is mounted on). As well, image differencing will not work for the few cases where the animal is completely still.

*Image enhancement*. Many off‐the‐shelf photo viewing systems now include various ways to adjust an image. Examples include contrast adjustment, saturation and luminance of particular colors, dehazing, sharpening, edge detection, etc. In the image analysis context, an analyst could apply various adjustments on a test image and—if effective at enhancing an entity's visibility—have that setting automatically applied when viewing other images.

#### 5.2.2 Timelapse example

Timelapse contains the first two methods above for enhancing the noticeability of small entities, both based on analyzing the differences between images. Timelapse does not include other image enhancement methods, but they could be added easily.

**Figure 5:**
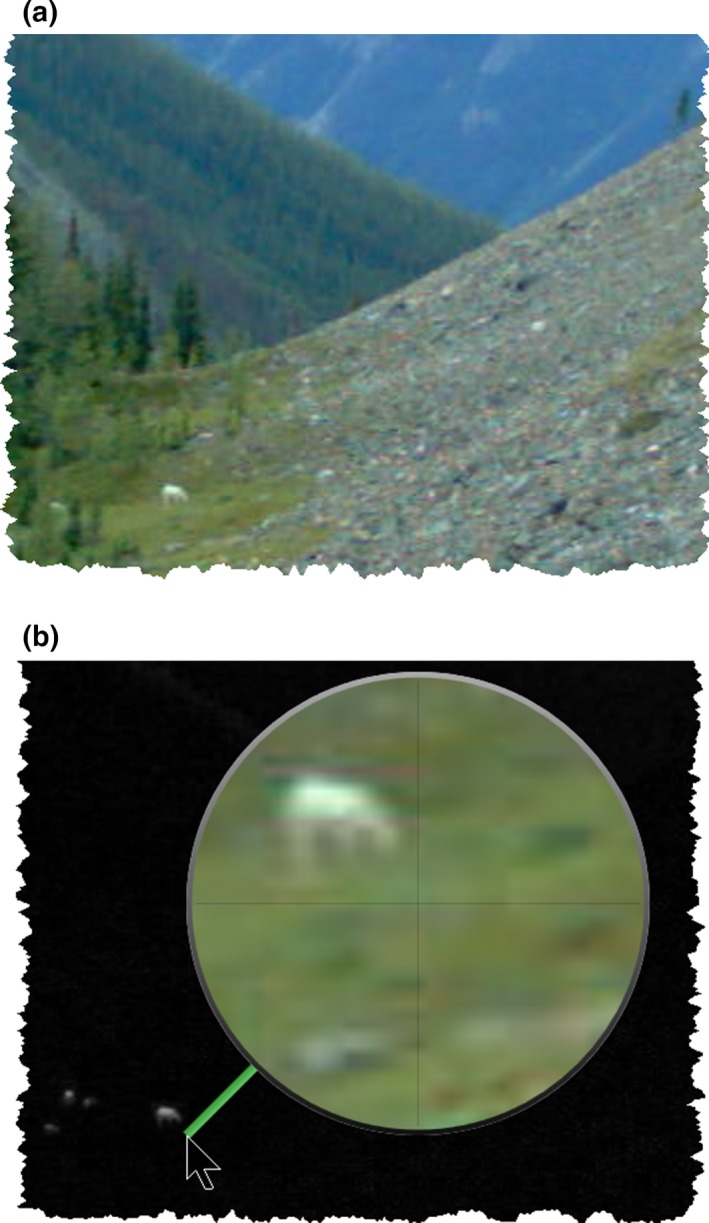
Image differencing. The analyst flips between the normal and differenced view of the image. (a) Normal image (cropped). Several goats are in the lower right corner, but the analyst may easily miss them. (b) Differenced image (same cropped region). The analyst investigates the white blobs with the magnifying glass, and sees that they are goats.

1. **Animation through rapid image switching.** Timelapse lets the analyst rapidly switch between the current image of interest and the next or previous image (using the arrow keys), where images are displayed immediately and without flicker. The analyst perceives this as an animation, where the differences between images—such as a small moving animal—“pop out.” Furthermore, the magnifier and zoom/pan level are maintained at their current setting and position during image switching, which helps the analyst spot differences in a magnified region.
2. **Image processing through image differencing** compares the original image to the previous image, the next image, or to both. The analyst toggles between the differenced and original image with a single key press. When blobs of interest appear, the analyst can use the magnifying glass (which displays that region in its original form) to investigate. Alternately, the analyst can use rapid image switching to see whether the blob has moved. Figure 5 provides an example of how this appears in practice. Figure 5a displays the normal image: The small goat can be easily missed or mistaken for a rock. Figure 5b is the differenced image: not only is the goat highlighted as a blob, but another goat partly hidden in the trees on its left is revealed. Figure 5b also shows the analyst furthering inspecting a blob via the magnifier, which displays the goat as it appears in the original image.

### 5.3 Problem—Entities within images may be difficult to see due to poor image fidelity

Because cameras are positioned in the field, the quality of the images produced can be compromised by many factors. Weather is one factor, where fog, rain, and snow can limit what is visible, especially at a distance. Lighting is another fact, such as sharp shadows mixed with bright sunshine, or failing light due to dusk and night‐time shots. The camera itself can be compromised, such as by moisture on the lens, or by focus problems.

#### 5.3.1 Design pattern: Enhance images whose fidelity is compromised

Various image processing techniques can enhance the clarity of compromised images, albeit with limits. Indeed, the previously described techniques used to enhance the noticeability of small entities could perhaps help here: contrast adjustment, color correction including saturation and luminance, sharpening, edge detection, etc. Dehazing will likely be of particular value in mitigating fog effects. As before, an analyst could apply various adjustments on one image and have that setting automatically applied to other similarly compromised images.

#### 5.3.2 Timelapse example

Timelapse does not yet include these image processing capabilities. Currently, the analyst would have to correct the image outside of Timelapse (e.g., using the many tools available in photograph editors such as Adobe Photoshop or Adobe Lightroom). The modified saved image would then be visible within Timelapse.

## 6 ISSUE: NAVIGATING IMAGES

Analysts are often tasked with inspecting tens and even hundreds of thousands of images in an image set. Thus reviewing, searching, and navigating between images should be rapid.

### 6.1 Problem—Tedious image navigation and review

An analyst may want to rapidly navigate and review a sequence of images for various reasons. She may want to scan all images quickly before coding them, in order to get a sense of what is in them. She may want to quickly move over “empty images” (e.g., scenes with no wildlife in it) until she spots an image containing something of interest. She may want to visually search the image set for a particular scene, for example, an image with wolves and cubs. She may also want to search for a particular image by its file name. The problem is that image packages often differ considerably in how they support navigation, where some navigational methods can interfere with the analyst's task. For example, image review would be severely impeded if each image has to be separately opened in its own window.

#### 6.1.1 Design pattern: Provide tools that allow rapid navigation and review of images

Analyst often examines images sequentially to see how they unfold over time. Stepping forward and backward through them should be visually instantaneous and should require minimal effort, for example, via a single key press or mouse click. Because image sets can number in the hundreds of thousands, analysts should be able to move, scrub, and jump through images quickly, similar to how one can scrub through a video. To help the analyst visually review and compare images during navigation (such as to detect changes as discussed in the previous design pattern), display settings such as zoom levels and the image location on the screen should be kept constant.

#### 6.1.2 Timelapse example

Timelapse contains many navigation methods, each allowing rapid image review.

**Figure 6:**
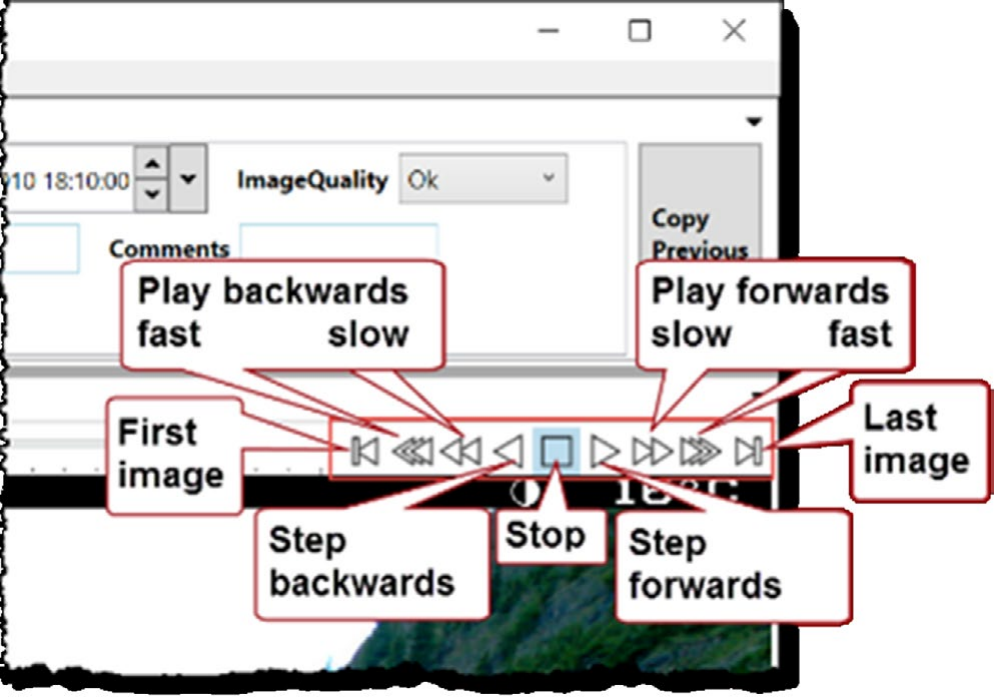
The Timelapse File Player

**Figure 7:**
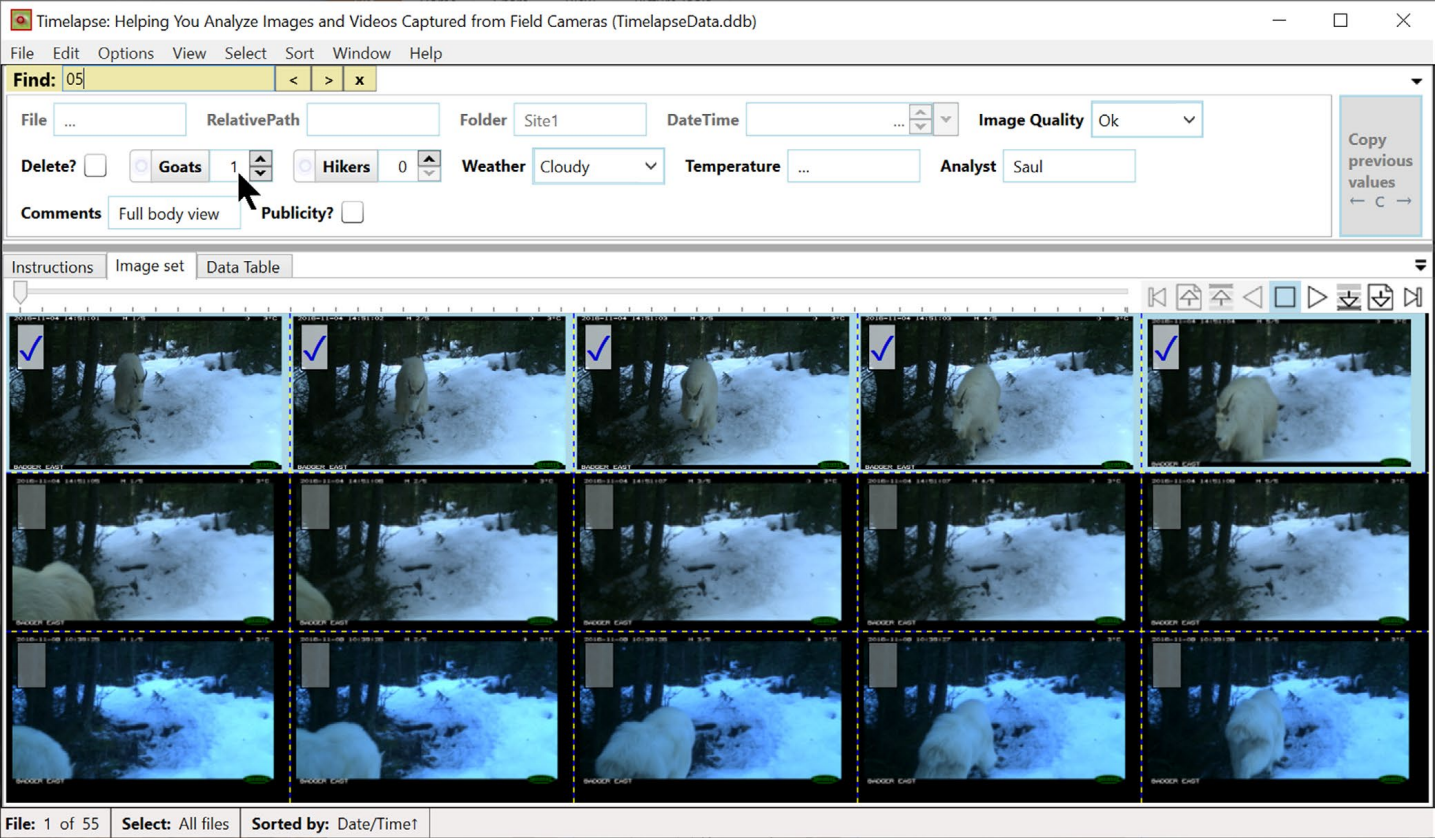
The overview showing selection and the Find feature

**Figure 8:**
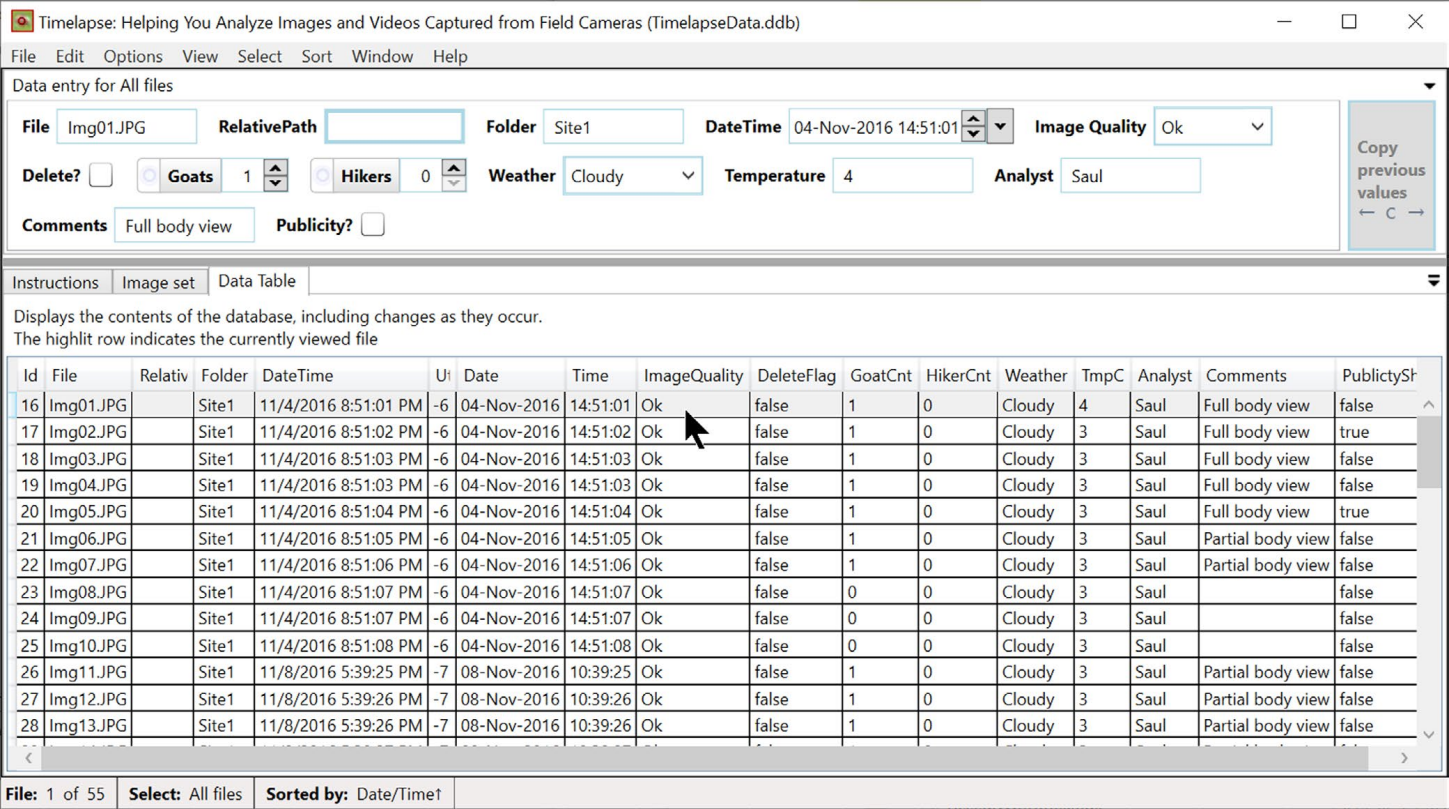
The Data Table view

1. **Forwards/Backwards controls.** Timelapse lets the analyst rapidly move either backwards or forwards between images via the keyboard (the arrow keys) or by the File Player (described next). Holding down the arrow key scrubs through successive images. Settings on the current image—the location of the magnifying glass, zoom and pan levels, image differencing (if any) are all retained during navigation, allowing the analyst to rapidly compare images for similarities and differences as he or she views them.
2. **File Player** (seen at the upper right of Figure 3 and annotated in Figure 6) provides an alternative mouse‐operated navigational control. Depending on the button pressed, the analyst can step through images, jump to the first or last image, or automatically play (and thus review) successive images at slow and fast speeds. These speeds are user‐configurable.
3. **Navigational Slider** (next to the File Player, see top middle of Figure 3) allows the analyst to both scrubs through and to rapidly jump across many images. Intervening images are displayed as fast as possible as the analyst moves the slider.
4. **The Overview.** Analysts can “zoom out” to see an overview containing multiple images, as illustrated in Figure 7. The more one zooms out, the more images are displayed, albeit at progressively smaller sizes. The analyst can navigate to a full‐sized view of a desired image (as in Figure 3) by clicking its thumbnail in the overview. The behavior of the navigational controls described above is also transformed to work with the overview. For example, the File Player controls now allow the analyst to navigate through successive images one by one, row by row, or page by page. Using the overview, the analyst can navigate and review collections of multiple images quickly.
5. **Find Search Bar,** illustrated at the top left of Figure 7, is somewhat similar to search bars seen in text editors. The analyst uses it to find and display the next file in the image sequence whose file name partially matches the entered text. Figure 7 illustrates a search for any file name containing “05.” Find works in both the single image view (Figure 3) and in the overview where the found image becomes the first image in the displayed image array (Figure 7). Find also works on suffixes. For example, if an image set is interspersed with video files, searching on “.avi” will step through all videos.
6. **Navigating via the data table.** Analysts have the option of a database view, which displays all the data entered so far as a scrollable table. This is available through the “Data Table” tab as seen in Figure 8. Each row represents all the data currently associated for an image. The analyst can inspect the rows for data of interest and click on that row to navigate to and view the image associated with that row (akin to the display in Figure 3).

## 7 ISSUE: ENTERING DATA

Our example data schema illustrated in Figure 2 and composed as a data entry interface in Figure 3 has relatively few data entry fields. This contrasts with the actual number of data fields that analysts can encounter in practice. For example, one of the agencies using Timelapse composed and regularly used a template defining 30 separate data entry fields that analysts had to fill in. Even if only a subset of those fields relevant to a particular image had to be filled in, data entry can quickly become tedious, error prone, and very time‐consuming when done over hundreds of thousands of images.

### 7.1 Problem—Typing is time‐consuming and error‐prone

Filling in data fields by typing is tedious. Fields have to be navigated, and typing takes time. Mis‐typing is common and introduces errors and inconsistencies in the data.

#### 7.1.1 Design pattern: Data entry controls should minimize or eliminate typing when possible

Selection (via the mouse or via tab/select/enter) should replace typing whenever possible. Since much data entry is repetitious, previously typed‐in entries should be offered as candidates for selection rather than requiring re‐entry.

#### 7.1.2 Timelapse example

**Figure 9:**
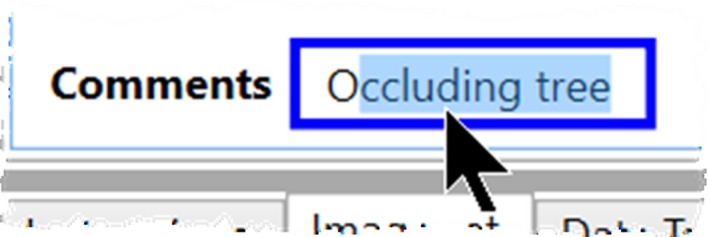
A note displaying a text prediction

Several data controls available through Timelapse (e.g., see top of Figure 3) favor selection via the mouse or through the keyboard's tab and arrow keys. An analyst selects a Flag's true or false value by clicking on its checkbox. She selects from a Choice's limited possibilities via a pop‐up menu. She can fill in Counters by clicking its up/down arrow buttons, or by clicking an entity in the image to count it (discussed shortly). She can edit the dates and times in the DateTime control by its up/down arrow buttons, or by directly selecting a date from a calendar. She can accept text predictions in Notes instead of typing an entry in full. Each Note tracks all previously typed text entries and uses those to predict the rest of the text as the analyst types. For example, Figure 9 illustrates the text prediction that appears after the analyst has typed the single letter “O.”

### 7.2 Problem—Counting is difficult and error‐prone when there are many countable entities present in an image

As previously discussed, some cameras are positioned to capture a wide field of view. In turn, the resulting images can contain many entities, perhaps of different types, that must be counted (e.g., Figure 1b). A wildlife monitoring example is a herd of animals present in the field of view, while a fisheries example is many anglers and nonanglers present on a popular lake's shoreline or in boats (Greenberg & Godin, 2015). All entities must be categorized and counted. The problem is that mis‐counting is easy. Common errors include losing track of the current count number, double counting that counts an entity more than once, and omission errors where an entity is accidentally skipped.

#### 7.2.1 Design pattern: The system should allow one to visually mark the entities present in an image that have been counted along with its type

Visually marking entities as the analyst counts them can mitigate common counting errors: the analyst can discern what has been counted and what is yet to be counted. If different entities are present and being counted, the visual mark could also indicate how that entity was identified. Visual marking also affords validation, where a (perhaps different) analyst can later review the image and its marked entities for counting or classification errors.

#### 7.2.2 Timelapse example

**Figure 10:**
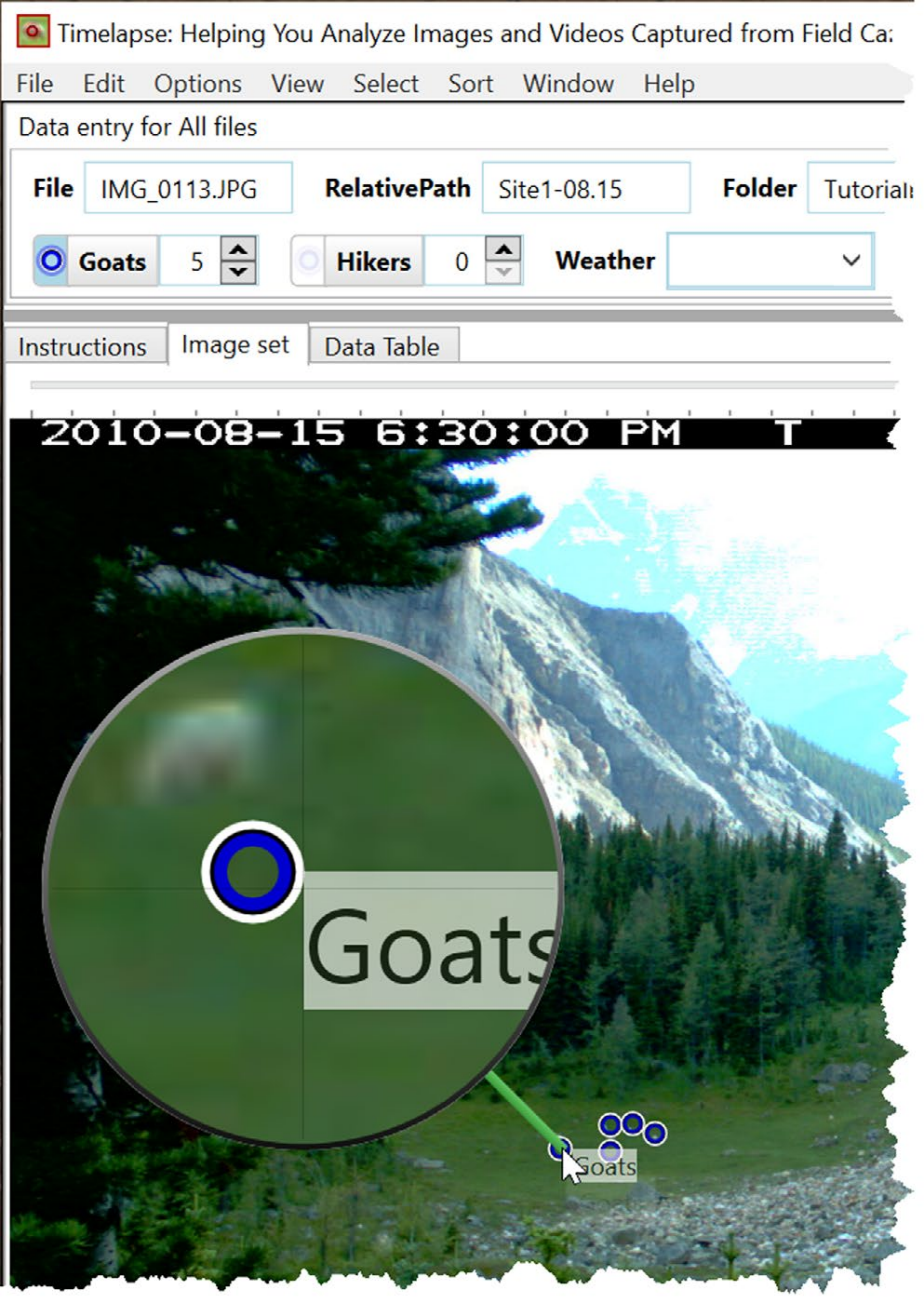
An activated Count control showing the visual marks next to the counted goats

The Counter data entry control supports interactive counting and visual marking and is illustrated in Figure 10. Here, the analyst has activated the “Goats” interactive counting mode by clicking its radio button. The analyst then counts goats simply by clicking next to each one: each click increments the count and adds a colored marker at that spot. Markers also work with the magnifying glass, where the analyst can inspect entities before marking them. Finally, markers provide feedback as to which Counter button they are associated with. For example, hovering over a marker reveals that it was counted as a “Goat” (as in Figure 10). Conversely, hovering over the Goats Counter button will highlight only those marks in the image counted as a “Goat.”

### 7.3 Problem—The analyst has to manually re‐enter image data even when it is available in a computer‐readable form

Analysts find it particularly frustrating when they have to re‐enter information that is already available electronically. This problem usually arises when software does not try to read in that information, or cannot make sense of that information without some guidance.

#### 7.3.1 Design pattern: The system should automatically fill in data fields if the information is available

The system should try to automatically fill in useful and readily available known information. This can include “standard” information such as file names, file location in folder, and the date and time the image was taken. As well, image files typically contain embedded metadata that describes attributes of the image, where some of these fields could be of interest and automatically imported. Yet metadata introduces its own problems. Most camera vendors embed a mix of standard and nonstandard (proprietary) metadata, which means that the information available is highly camera‐dependent. For example, some may include ambient temperature and GPS location of the station, but others may not. Another issue is that different venders may name fields differently, for example, the outside temperature may be recorded in one camera as “Ambient temperature,” and in another as “Temperature C.” Thus, the analyst should be able to specify what metadata fields of interest, if any, should be imported, and where that information should go.

#### 7.3.2 Timelapse example

Timelapse automatically fills in data fields in two ways.

**Figure 11:**
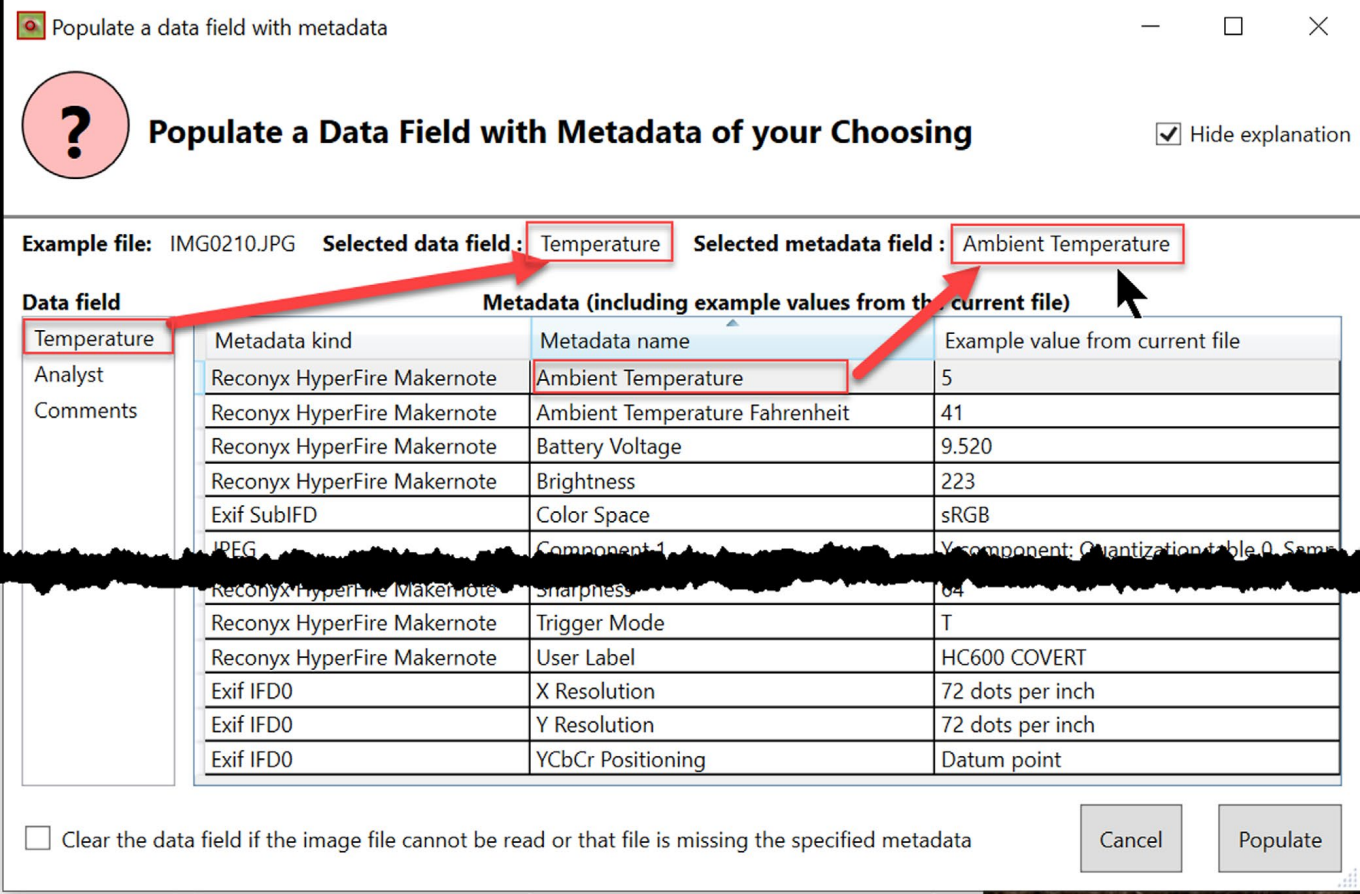
The metadata inspector. The analyst can see what metadata fields are available, and link a particular metadata field to a Timelapse data field to import the metadata value into that field across all images

1. **Standard file information.** Timelapse template schemas always include several default data fields representing standard file information: its name, its location (as a folder name and relative path), and the date and time that image was taken (as a combined Date/Time Field). Figure 2, top, shows these data fields in the top rows: The grayed out cells are not editable. When the analyst first invokes Timelapse on an image set, Timelapse scans every file for that information and fills in those corresponding data fields.
2. **Metadata.** Timelapse includes a *metadata viewer*, which the analyst can invoke on one of the images being analyzed and specify what data should be imported. We explain how this works by the example illustrated in Figure 11. The metadata viewer displays all the metadata found in the image as a table. The analyst sees, in the first row of the table in Figure 11, that the camera has recorded some metadata of interest: the “Ambient Temperature” field that records the temperature at the time the image was taken. As annotated in red in Figure 11, the analyst can link the Ambient Temperature metadata field to a Timelapse data entry Note field—in this case a field called “Temperature”—simply by selecting both of them. When the analyst clicks the “Populate” button (bottom), the “Temperature” field for each and every image is automatically filled in with the Ambient Temperate metadata value recorded in each image. The process can be repeated for other metadata of interest.

### 7.4 Problem—The analyst has to enter information that the computer should be able to recognize by image analysis

Analysts usually have experience using a variety of other image‐based systems when doing day to day and recreational tasks. Many include capabilities that recognize aspects of an image, with perhaps face recognition, bar code reading, and text recognition being common examples. Analysts may find it frustrating to enter data that they believe could be detected through image analysis and automatically filled in.

#### 7.4.1 Design Pattern. The system should, if plausible, use image analysis techniques to automatically fill in data fields

Generally speaking, image analysis is the extraction of meaningful data from a digital image. One form of image analysis is image recognition, where complex algorithms use models built upon prior human classification to identify features in an image, such as objects, people, text, faces, and so on. As previously discussed, various researchers are now applying image analysis, and in particular image recognition techniques, to classify images from camera traps. A typical objective is to see how well various recognition algorithms identify animal species (e.g., Norouzzadeha et al., 2018; Schneider et al., 2018; Tabak et al., 2018; Yousif et al., 2019), and even in recognizing individuals in particular species (e.g., Cheema & Anand, 2017; Crouse et al., 2017). Simpler image analysis methods can also identify other image aspects, for example, differentiate between color versus monochrome images, light versus dark images, and so on. Because image analysis and recognition are not yet full proof, manual verification of the data will be required, at least for the near future. Thus, extracted data should be integrated into the analyst's workflow in a manner that allows the analysts to check and correct that data as needed.

#### 7.4.2 Timelapse example

**Figure 12:**
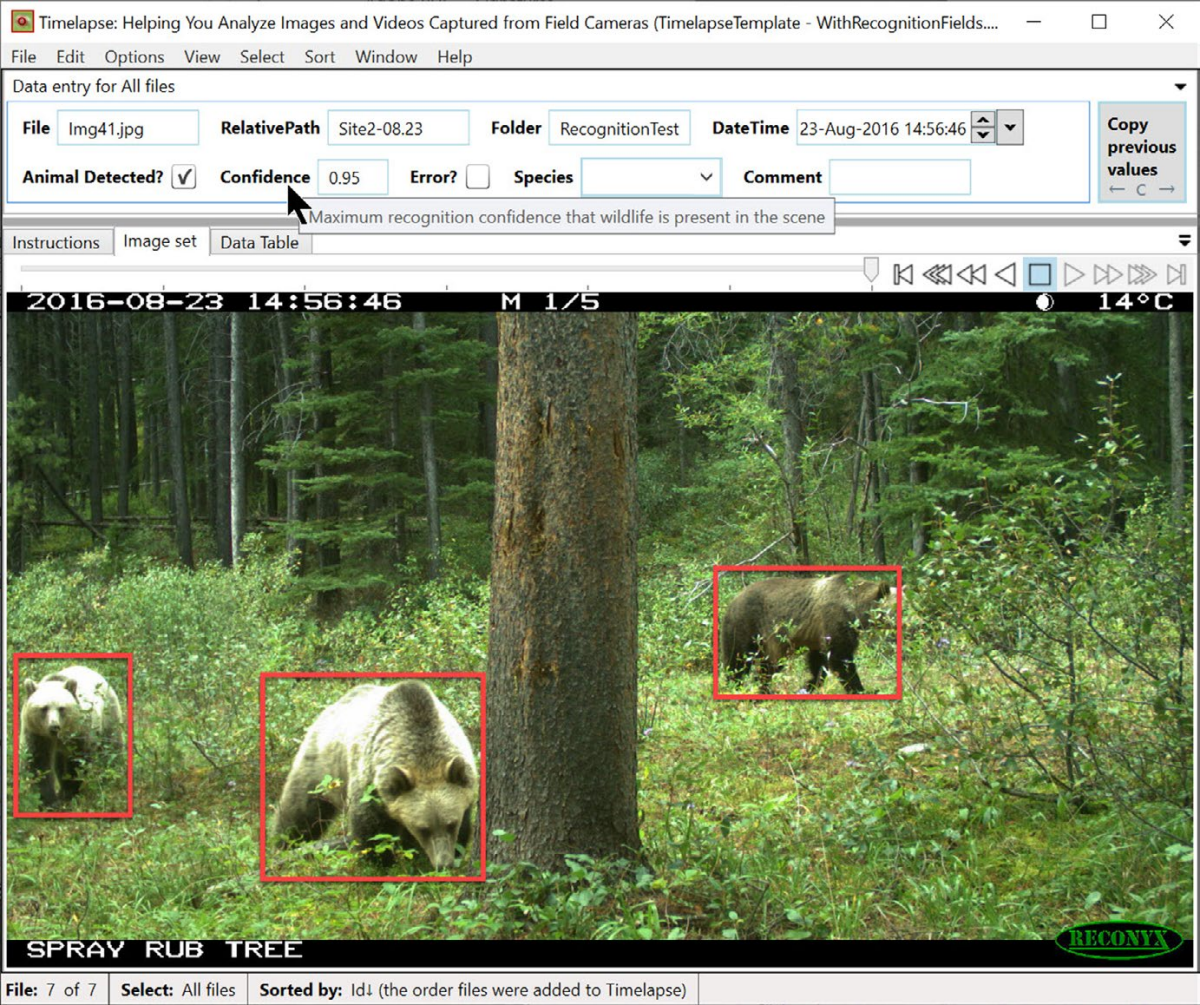
Timelapse prototype incorporating recognition data: bounding boxes are drawn around each suspected species in the image when its detection confidence exceeds a user‐defined threshold

1. **Dark images.** Some of the agencies we worked with used cameras set in Timelapse mode that periodically took images over a day's 24‐hr period. A good number of those images proved of little value because they were too dark (e.g, shots taken at night time) and added clutter to the images being reviewed. To help identify overly dark images, Timelapse incorporates an image analyser that automatically classifies images against a user‐configurable darkness threshold. Its classification is recorded in the “Image Quality” data field of every image as either “Dark” or “Ok.” Timelapse also includes the ability to filter the displayed images by its data, which we will discuss shortly. Analysts could apply a “Dark” image filter to display only dark images, where the analyst can quickly review and correct the classification if needed, and perhaps discard those night‐time shots. Alternately, the analyst could apply an “Ok” filter, which displays only the nondark images.
2. **Animal detection and recognition.** We are currently working with several vision researchers who specialize in automated animal detection (e.g., whether an animal is in an image) and species recognition (which species the animal is). (Microsoft, 2019; Schneider et al., 2018). Figure 12 illustrates a Timelapse prototype that imports and displays animal detection data produced by Microsoft's “Megadetector” model (Microsoft, 2019). Basically, Megadetector scans all provided images and outputs data to a file. For each image, Megadetector detects whether an animal, person, or vehicle is in an image, its confidence of correctness, and the coordinates of a bounding box outlining each entity's location. Timelapse imports that data and draws a bounding box atop each identified entity above a detection confidence threshold (set by the analyst). The analyst then uses the standard Timelapse features to select detected entities and review predictions at given confidence levels and accepts or rejects those predictions as needed.

### 7.5 Problem—Cameras often record incorrect or ambiguous timestamps

We have observed many issues resulting from the way camera traps record date and time. While the software can automatically import and fill in date/time fields, analysts may have to correct those after the fact. The problem is that it is incredibly time‐consuming to manually correct every image's date and time. Common issues we have observed are as follows:

- The camera is not set to the correct date and time when deployed, meaning all date/times are off by a fixed amount.
- The camera does not take into account changes in daylight saving time, which means a large subset of images are off by an hour.
- The camera's internal clock drifts, for example, it runs slow or fast, which means that the date/time of successive images is increasingly inaccurate.
- The camera records dates ambiguously. For example, consider a date recorded as 02/10/2019. This date can be interpreted as either October 2, 2019 in day/month order, or as February 10 in month/day order. Even worse are cameras that record the year as only the last two digits, for example, 02/10/10 could be interpreted many different ways. This issue is exacerbated by the way different countries set different format standards for encoding dates and times (e.g., see Wikipedia: Date format by country).

#### 7.5.1 Design pattern: The system should provide facilities to bulk‐correct common date/time errors

All the above errors care amenable to bulk‐correction, albeit with some manual guidance. For example, if the camera was not set to the correct date and time, the analyst would only have to enter the correct date for the first image. The system could calculate the difference between the two, and then use that difference to time‐shift the date and time for all subsequent images. Similarly, the analyst can specify where the daylight savings time change should occur and time‐shift previous or subsequent images by plus or minus an hour. To correct for internal clock drifts, the analyst can specify the correct time for the last image, where the system would then calculate a drift factor and adjust the times across all images. When the software detects a possibility for ambiguous dates, it can notify the analyst who can then indicate which date format to apply.

#### 7.5.2 Timelapse example

**Figure 13:**
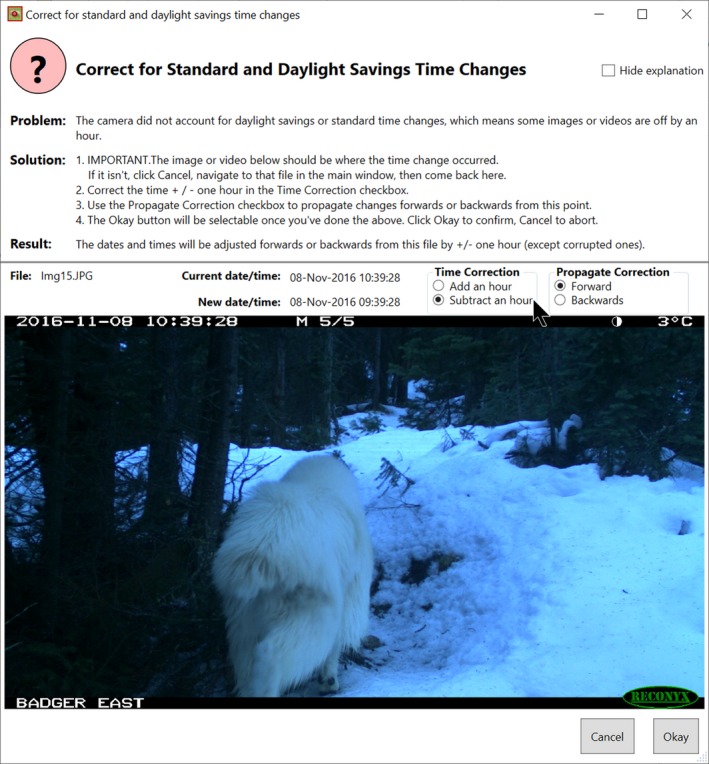
Dialog for correcting daylight savings time

Because we expect analysts to have to correct dates only infrequently, analysts can raise specialized dialogs for each type of date/time error mentioned above: each dialog includes full instructions and an easy to use interface for specifying how the date should be corrected. For example, Figure 13 illustrates the Timelapse dialog for correcting standard/daylight savings time errors. The analyst navigates to the first image that should be corrected, and then specifies (via various checkboxes) how the correction should be applied. A preview of the corrected date and time is also displayed.

## 8 ISSUE: ENTERING REPETITIOUS DATA

### 8.1 Problem—Similar data are often entered and re‐entered over many images

Image sets often comprise subsets of very similar images. For example, a motion‐triggered camera may capture a sequence of multiple images of an animal moving through a scene. As another example, an image set can comprise a small set of recurrent but interspersed images, for example, images containing goats, or elk, or deer, or nothing at all. The data entered that describe these images are often highly similar. Even when the analyst recognizes these similarities, she still has to manually enter the same data per similar image over and over again. This leads to highly repetitious and very time‐consuming data re‐entry.

#### 8.1.1 Design pattern: It should be easy to re‐enter data previously entered elsewhere

Various general techniques are known in other domains for re‐entering the same data efficiently. Examples include history lists, copying and pasting, predictions based on previous entries, data propagation, and others.

#### 8.1.2 Timelapse example

Timelapse includes several techniques for easing the task of entering repetitive data across multiple images.

**Figure 14:**
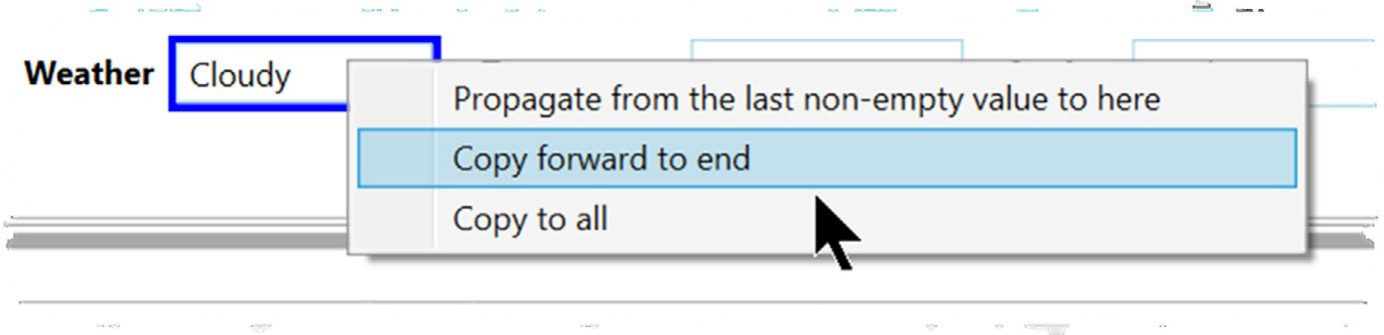
The data field's pop‐up menu for propagating data

**Figure 15:**
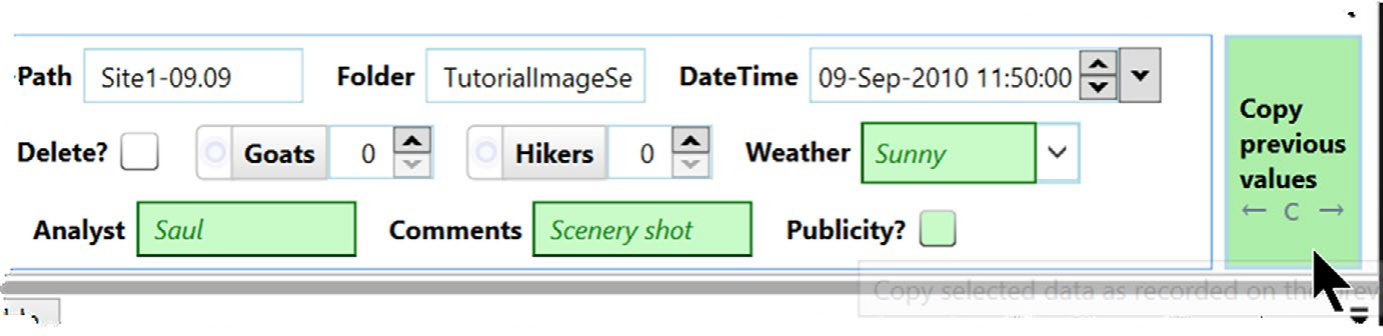
Copy Previous Values button, showing previews of the data to be copied

**Figure 16:**
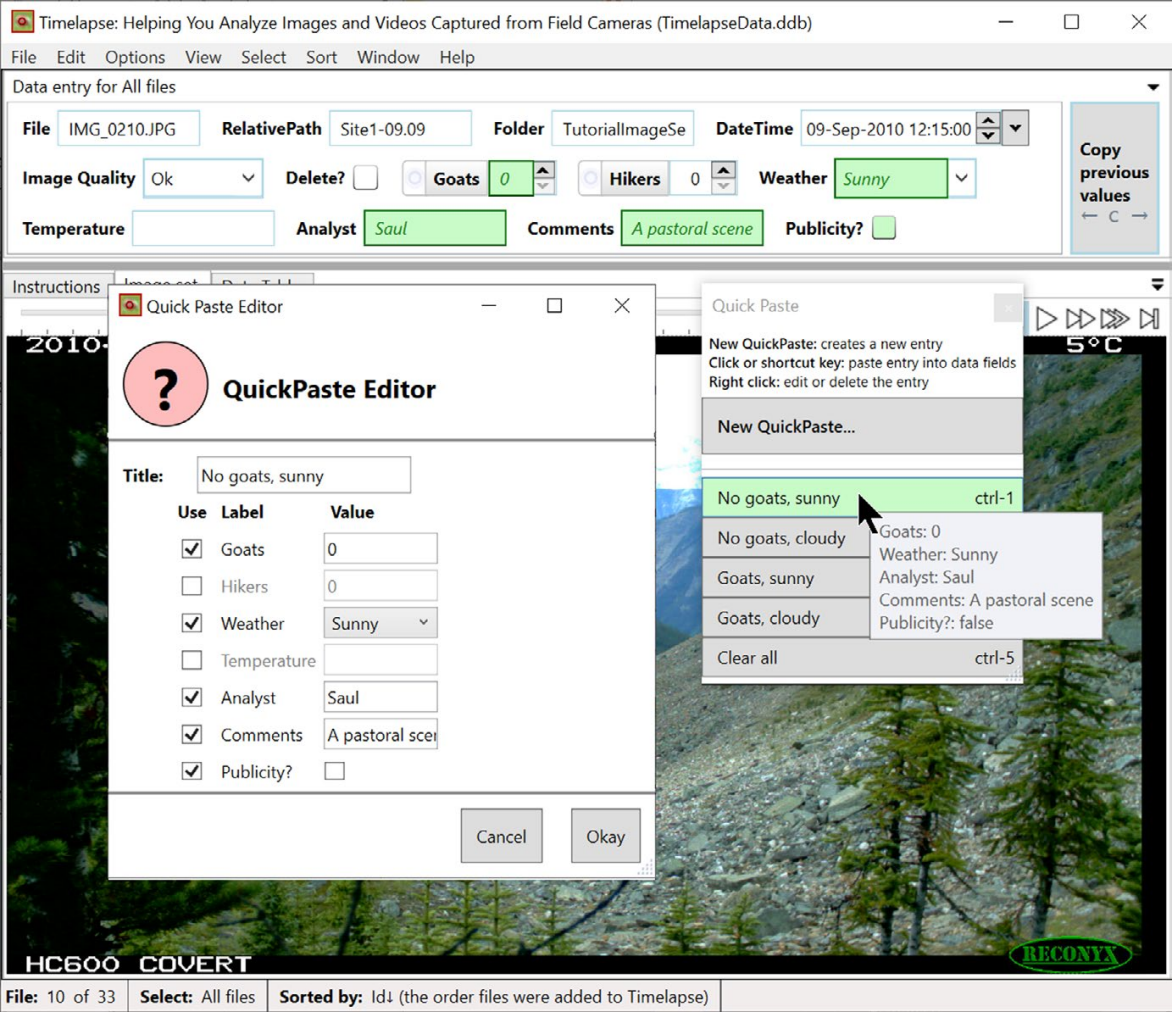
The QuickPaste editor (left) and the QuickPaste window (right)

1. **Text prediction in a single data field.** As already discussed, Notes include auto‐completion capabilities. They maintain a history of previously typed text entries and use those to predict the rest of the text as the analyst types.
2. **Propagating data across a single data field.** Every data field includes a pop‐up menu that allows the analyst to propagate data across a sequence of images (Figure 14). *Propagate from the last nonempty value to here* uses back‐filling. That is, it will copy the last nonempty value entered by the analyst in a data field (e.g., several images back in the sequence) to every intervening image up to the current image. For example, the analyst may enter an image's weather as “Sunny,” then navigate forward through the images until the weather changes, and then backfill the intervening empty fields with that value. *Copy forward to end* is somewhat similar, except it forward‐fills the current value to all remaining images in the sequence. It can be re‐applied at any time where it over‐writes existing values. For example, an analyst may Copy forward Cloudy (as in Figure 14), then move through the sequence until the next non‐Cloudy day is noted, enter the new value, and then Copy that forward as well. *Copy to all* copies the current value to all images. For example, the analyst may just enter their name once in the “Analyst” field and copy that to all images.
3. **Copy Previous Values.** Image sets often contain runs of identical images, where some of the data entered over the next image are identical to what was entered in the previous image. Timelapse supplies a “Copy Previous Values” button, illustrated in Figure 15. Pressing this button copies the previous image's values from particular data fields (those set as “Copyable” in the template: See Figure 2) to the current image's data fields. As illustrated in Figure 15, previews of what fields are affected and the data that will be copied are displayed and highlighted in green when the analyst hovers the mouse over the Copy previous values button.
4. **Quickpaste: Copying and pasting multiple data fields.** Analysts typically recognize when they entering a small set of similar data patterns over and over again. Timelapse provides QuickPaste as a way for the analyst to capture and name these data entry patterns, where the analyst can then paste that pattern into an image's data field via a single mouse click. Figure 16 below illustrates this through a simple example. The analyst has raised the QuickPaste editor (left) to compose a QuickPaste entry: she has titled the entry “No goats, sunny” and has selected and filled in which data fields should be used (Goats, Weather, Analyst, Comments, Publicity) and the values to be pasted. This entry is then added to the list of other QuickPaste entries in the QuickPaste window (right side). The analyst can then use the QuickPaste window to select and paste particular patterns into the image's data entry fields. As illustrated in Figure 16, when she hovers over an entry, a preview of the values to be pasted appears in the affected data fields (highlighted in green). Clicking the entry pastes, those values into the field. While requiring some initial setup to create these custom entries, QuickPaste becomes a very effective and efficient way for entering common data patterns.

### 8.2 Problem—Reviewing and entering repetitive data image by image can be inefficient

Most image packages display a single image at a time, where the analyst has to inspect and enter data for them individually. Bulk‐image inspection and data entry are not possible.

#### 8.2.1 Design pattern: Allow the analyst to inspect and bulk‐enter data for multiple images at a time

The system should provide facilities for displaying multiple images at a time (e.g., a table of large thumbnails). The analyst should be able to select particular images with common features, and then bulk‐entering data for those selected images all at once. The analyst should also be able to choose the appropriate thumbnail size, as the features of interest need to be discernable.

#### 8.2.2 Timelapse example

The overview supplied in Timelapse, discussed above and previously illustrated in Figure 7, allows the analyst to review multiple images at the same time. The analyst can quickly trade‐off the number of images displayed versus the image size (to optimize just‐discernable features with the number of images shown) by zooming in or out of different overview levels with the scroll wheel. The analyst can then select and bulk‐edit data for one or more of those images. For example, Figure 7 shows how the analyst selected only those images with a full view of a goat in it (the first five images), where she has entered a “1” in the Goats Counter field and “Full body view” in the Comment field. Those values are then applied to all the selected images. Interface subtleties are also addressed. As multiple selections are done, the data fields and their contents are adjusted to reflect that selection. For example, and as also shown in Figure 7, the DateTime data field is disabled as bulk‐editing that field makes little sense. If a data field in the selected images all share the same data value, that value is displayed. Otherwise a “…” symbol is displayed to indicate that their values differ.

## 9 ISSUE: SORTING AND FILTERING THE IMAGE SEQUENCE

### 9.1 Problem—Images are often presented in a single sort order, usually based on their file name, which may not reflect how the analyst wants to view them

Analysts usually inspect images as a sequence, one after the other. Thus, the way images are ordered (sorted) can affect what they see and how they interpret images as events unfolding over time. Consider the example of a motion‐triggered camera taking images of one or more animals moving through the scene. If the presentation sequence is in time order, the analyst will recognize that those images relate to one another, as they are capturing a single event. As another example, the analyst may wish to review already classified images ordered by a combination of criteria. For example, the analyst may want to get a sense of whether the number of goats using the pasture in Figure 3 is correlated to weather conditions. This can be done by ordering images by weather and then by the number of goats. The problem is that most systems typically order images only by its file name and do not allow any other sorting capabilities.

#### 9.1.1 Design pattern: Allow the analyst to sort images by one or more criteria

Providing the ability to sort by date/time rather than file name is perhaps the most fundamental sort capability that should be included. While cameras typically add a sequence number to a file name as images files are created, there is no guarantee that they will be presented in time order for example, alphabetically sorted files named 1.jpg, 2.jpg… 10.jpg would be presented as 1.jpg, 10.jpg, 2.jpg…, which breaks time ordering. Ideally, the software will also allow the analyst to sort on any data field or combination of fields and their data values.

#### 9.1.2 Timelapse example

**Figure 17:**
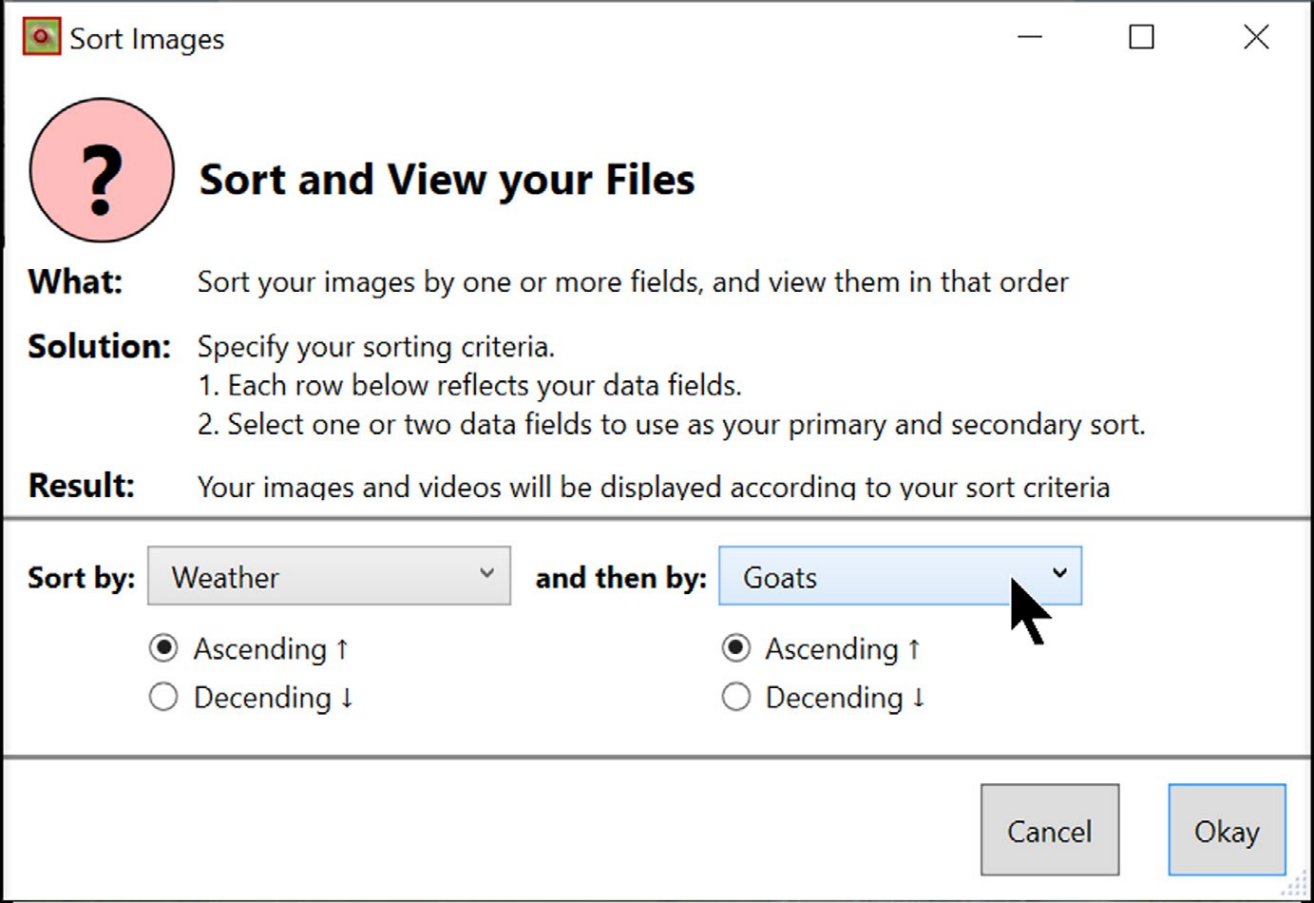
The sorting dialog

Timelapse provides a sorting capability based on one or two data fields of the analyst's choosing. The analyst can quickly select (via a menu) common sorting criteria including image load order, date/time order, how images are organized into folders, and by particular data entry field contents. The analyst can also raise a custom sort dialog (Figure 17 below), where she can choose primary and secondary sorting criteria from a drop‐down menu that lists labels for the data fields. In this case, she is sorting by weather and then by Goats. Images are then presented in that sort order. The rows in the data table in Figure 8 are also updated to that sort order.

### 9.2 Problem—The analyst may need to view a particular subset of images

Analysts may, at times, be interested in only a subset of the available files. Yet finding and viewing the images in this subset can be problematic, especially with large image sets comprising tens of thousands of files. As one example, the analyst may want to verify and possibly correct prior image classification category, for example, that all system‐classified dark images are indeed dark, that images classified by another analyst as “Goats” all contain goats, and so on. As another example, the analyst may be interested in only those files taken at a certain site and between particular dates. As yet another example, the analyst may want to review a particular image classification in order to choose an archetypical image, for example, an excellent image of a goat to be used for publicity purposes.

#### 9.2.1 Design pattern: Allow the analyst to specify criteria that filters which images are displayed

The system should provide the analyst with a query facility and search engine. The analyst should be able to specify a search query, where the system filters images so that it only displays images matching that query. Query criteria should include queries against the values recorded in the image data fields.

#### 9.2.2 Timelapse example

**Figure 18:**
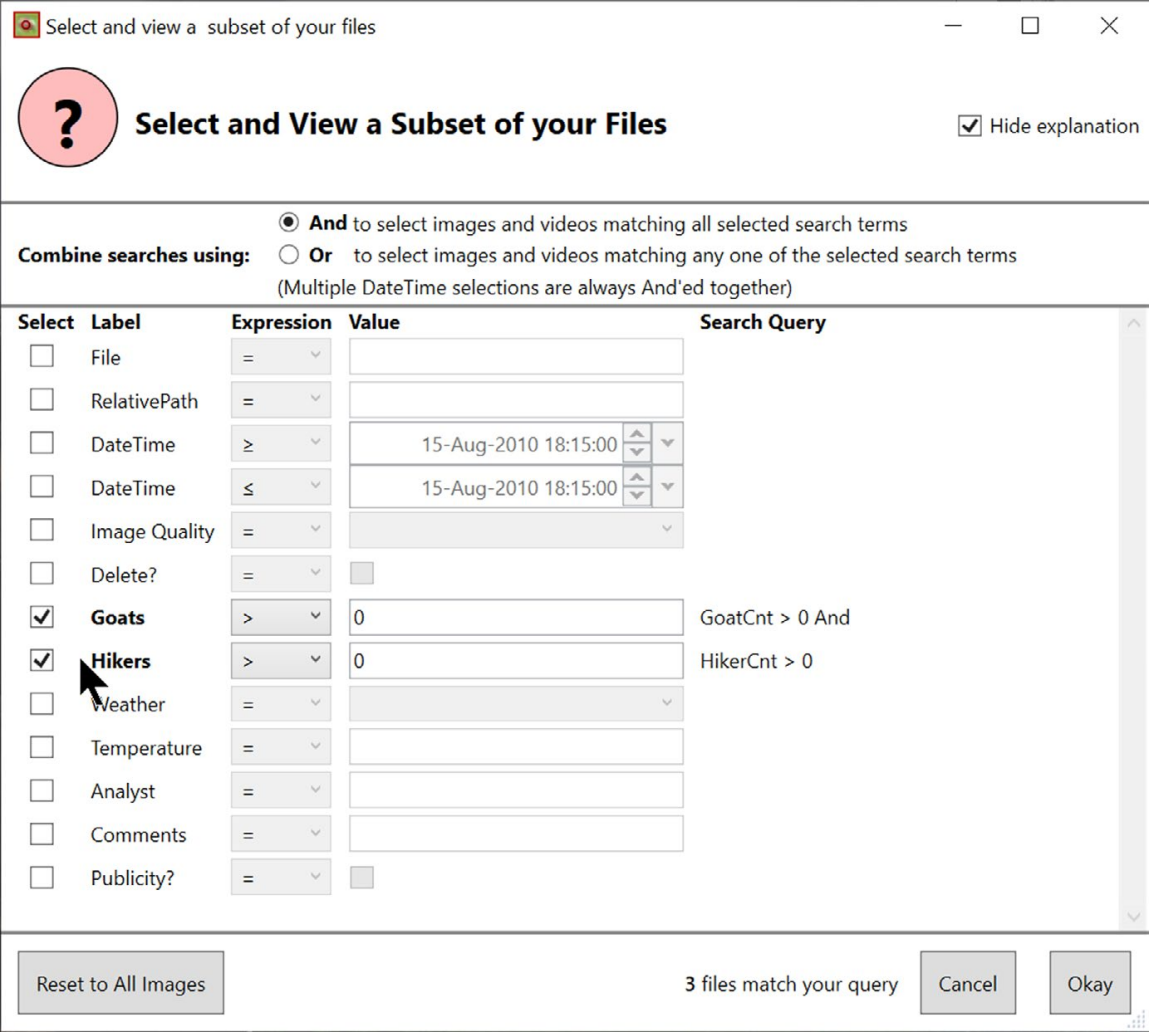
The query dialog for filtering images from view

Timelapse incorporates a free database (SQLite: http://www.sqlite.org) to store the data entered by the analyst. SQLite includes a query language for searching for matching records. Thus, Timelapse can perform any standard database search against that data, where search results are returned as records describing the matching images. Those images are then displayed. However, it is unrealistic to expect analysts to compose cryptic SQL query expressions. As a better alternative, Timelapse displays a dialog box listing all data fields, as illustrated in Figure 18. The analyst then composes a query by selecting the data fields of interest, and then specifying the values that should be matched. The system translates that into an SQL query and returns only those images that match the query. For example, in Figure 18, the analyst is interested in the interaction between goats and hikers and wishes to see only those images that have both a goat and a hiker in it. The analyst selects the Goats and Hikers data fields for use (the “Select” column on the left) and has specified that both have values greater than 0 (the “Expression column”). The “AND” checkbox at the top indicates that both those constraints must be satisfied. Feedback (bottom right) indicates that three files match that query. After clicking Okay, only those three images will be available for navigation and display. Had the analyst had clicked the “OR” checkbox instead, then all returned images would contain either one or more goats, or one or more hikers, or both. The Timelapse Sort function can also be applied to the results, for example, to show all images with both goats and hikers, but sorted by the number of goats and then by the number of hikers.

### 9.3 Problem—The analyst may need to consider images taken over a short time period as a unit

As previously mentioned, camera traps set in motion‐capture mode are often triggered when an animal or herd is moving through a scene. This can result in a burst of images that capture that activity, which we define as an *episode*. Episodes are sometimes treated differently than individual images. For example, we saw analysts manually determine which images fall into an episode (e.g., by examining their timestamp), count the unique wildlife seen in that episode, and enter that data into only a single image. They do this to avoid inflating the number of wildlife present. To illustrate, consider the analyst who has to count the number of hikers using a trail. A single hiker may appear on several images over time, perhaps due to motion triggering, or because the hiker is milling about in the camera's field of view. To avoid double counting, the analyst would only count the hiker once in this series. The problem is that it is laborious for the analyst to recognize which images belong together in an episode.

#### 9.3.1 Design pattern: The system should identify and group episodes of time‐related images

Various strategies can be used to identify episodes. For example, some cameras include metadata that indicate whether an image is part of a motion‐capture sequence, as well its position in that sequence (e.g.,1/5, 2/5, etc.). While useful, it is limited as an episode can easily comprise two or more back to back motion‐capture sequences. Alternately, a reasonable heuristic is to have the system examine the time interval between time‐ordered images. If the interval is small, the system would group them together as part of an episode.

#### 9.3.2 Timelapse example

**Figure 19:**
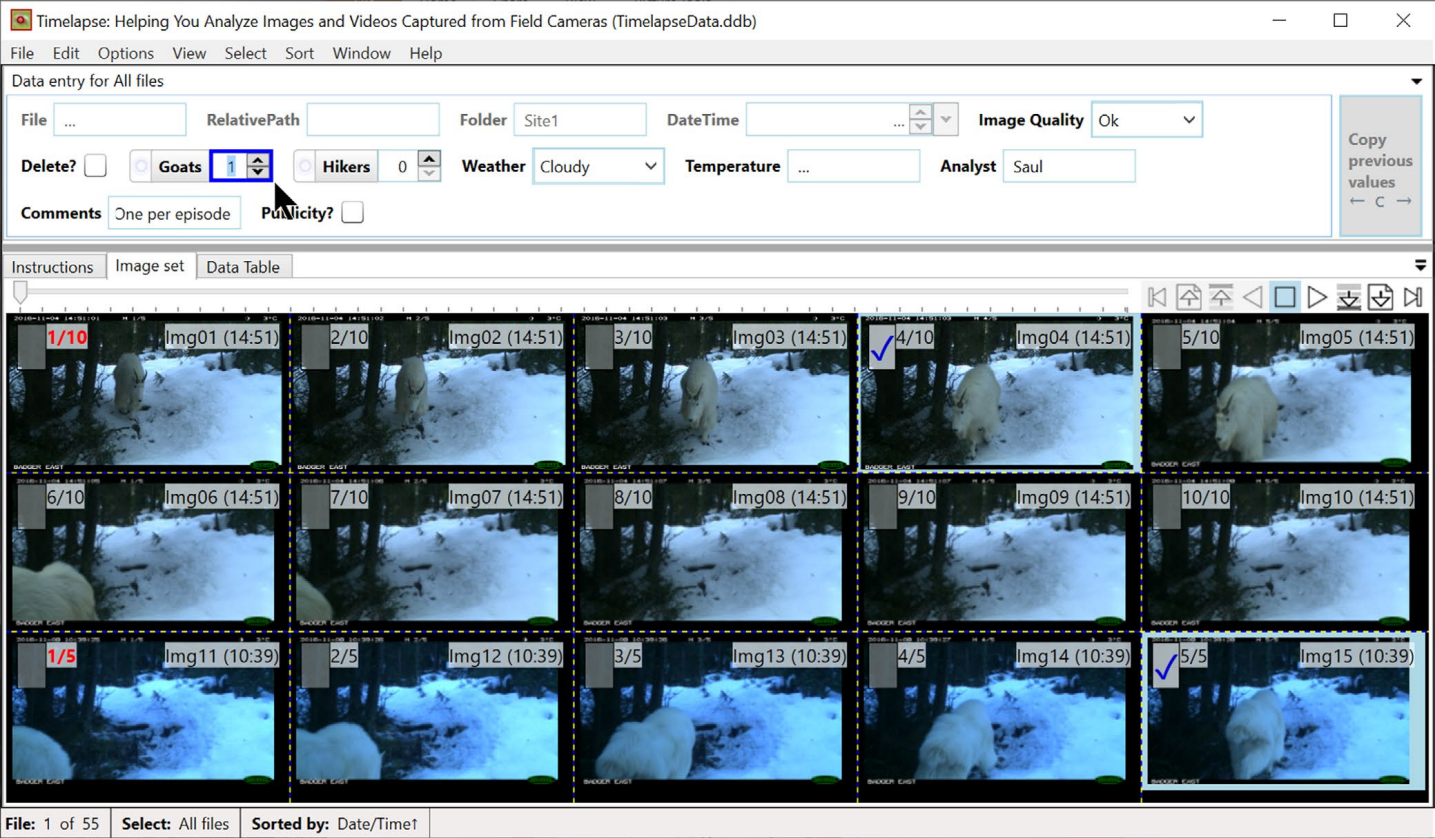
Episodes. Here, the analyst is using a strategy of entering data on only one image per episode

Timelapse uses the heuristic above, where the analyst can ask it to group together images separated by a small user‐configurable time interval. Timelapse then annotates each image to indicate how images relate to one another as an episode. Figure 19 is similar to Figure 7, except that it now illustrates how episode annotations appear in the overview. The first image in an episode is colored red (top left) so that the analyst can visually identify the start of the episode. That and subsequent images in the episode are given a sequence number (e.g., 1/3, 2/3, and 3/3). A timestamp is also overlaid atop the image, so that the analyst can examine the time differences between those images if needed. If an image does not belong to an episode, it is marked as “Single” (not shown). In Figure 19, the first ten and the last five images are identified as two different episodes of a goat walking through the scene. In this case, the analyst does not want to double count the same goat. Consequently, she selects the best image in each episode (Img04 and Img15), and increments the Goats counter of only that image.

## 10 DISCUSSION

Decisions on what software is used to inspect and encode image data have consequences on how well an analyst can perform their job. Yet, we question how some agencies make their decision. We have seen some consider only the stock software available on typical computers: For example, Microsoft Photo Viewer to view images, and an Excel spreadsheet for data entry. This is inefficient. For example, we previously studied how analysts entered data using spreadsheets versus an earlier version of Timelapse. Timelapse provided time improvements of ~200% or more, which translates into significant cost savings (Greenberg & Godin, 2015). We saw other agencies use either researcher‐based software or the stock software that came bundled with their cameras without considering the consequences of that choice on the analyst. Some agencies may also make their choice based on other factors, such as how the software stores data in a format amenable to standardization or later analysis versus how that data are actually entered by analysts. We advocate that decisions on which software is used should deeply consider how well they support the analysts' tasks. The design patterns described earlier should be part of that consideration. Poor system choices imply tedious data entry, are error‐prone (which affects the validity of the collected data), are morale‐sucking, and—in the long run—are very expensive in terms of analyst time.

While our design patterns mitigate various problems faced by analysts, we recognize that these problems range in seriousness, in frequency of occurrence, in applicability to particular projects, and in consequences if they are not addressed. We also recognize that our catalog of design patterns is just a starting point and future work is required: There are surely other problems and design patterns that could and should be articulated and considered in camera trap analysis design. For example, if image analysis is done through crowdsourcing and citizen science (Swanson, Kosmala, Lintott, & Packer, 2016), design patterns specific to that audience would likely emerge. Design patterns can also extend beyond interface features. For example, they can recognize and address the problems related to data management issues (e.g., Ivan & Newkirk, 2016), data validation, and data standardization and scaling across the field (e.g., Steenweg et al., 2017).

We also stress that design patterns are not “feature list.” Rather, each design pattern suggests a design approach that can be adapted, refined, and specialized to best fit the project, the background and needs of the analysts, and the equipment available. Each design pattern can also inform decision‐making. If the problem and design approach is relevant, that should become a factor influencing the requirements analysis of the software being developed or for a manager deciding between available software systems.

We also show how our own Timelapse system implements the design pattern. These are intended to serve as concrete examples rather than prescriptions. Of course, the specific techniques used by Timelapse could be implemented “as is” in other camera trap systems. However, we recognize—and indeed encourage—future system designers to see beyond our own solutions, where they should seek solutions that implement the design pattern in even better ways. For example, Timelapse was intentionally designed to work on lowest common denominator computers typically available to analysts: Microsoft Windows running a keyboard and mouse on a conventional low‐cost computer as found in many agencies. Thus its design eschewed more modern interaction techniques, such as touch interaction, as we felt it would limit its deployment. If a system such as Timelapse was redesigned to run on (say) a touch‐based tablet, we would expect different design solutions that still follow the above design pattern recommendations. Similarly, Timelapse was designed to work off‐line so analysts could work in the field on disconnected laptops. If Timelapse was redesigned to work as a networked client or over the web, design solutions would have to account for performance aspects such as network bandwidth and latency that could affect responsiveness and rapid image display.

As mentioned, we recognize that our list of design patterns is incomplete, where future work should elicit other design patterns to produce a comprehensive catalog. Researchers should continually conduct interviews and observation of analysts as they work to gain an even more nuanced understanding of their core tasks and problems. Because camera traps are broadly used for many quite different purposes, domain‐specific design patterns should be developed. Other software systems should be reviewed and compared for how they address problems and deliver solutions not covered by Timelapse, and whether those can be encapsulated as useful design patterns. As well, our design patterns are limited to only the analyst's interface for inspecting images and entering data. Future work should consider design patterns for other related tasks. One example concerns interface patterns that suggest how a project manager can view and manage data within and across projects. To illustrate, the Reconyx MapView software (Reconyx Inc, 2016) includes a map interface that lets the project manager or analyst geo‐locate study sites and stations onto it, and which lets them drill down into the captured data.

Finally, we recognize that elements of various design patterns are based on aspects well‐known within the field of human–computer interaction, information visualization, and experience design. These fields have a rich literature of research, practitioner's guides, and texts relating to the design of systems for human use, including methodologies that describe how to test how well a person can use that system and its features (e.g., Shneiderman et al., 2016; Spence, 2014). As well, various stock components and interaction techniques are readily available in software development tools, where most are based upon best practices of user interaction. The catch is that decisions of what is relevant must still be made on the needs of the domain being considered. This is the purpose of this paper, where it identifies problems and solutions as design patterns relevant to the domain of camera trap image analysis.

## CONFLICT OF INTEREST

None declared.

## AUTHOR CONTRIBUTIONS

See the Section 3, which describes the various roles played by the authors in more detail. Greenberg developed the Timelapse software and was the primary author of the design patterns listed here. Godin and Whittington contributed regularly to the Timelapse software design via on‐going discussions of its features and its use by their team of analysts. They also reviewed and contributed to various drafts of this paper.

### OPEN RESEARCH BADGES

This article has earned an https://openscience.com for making publicly available the components of the research methodology needed to reproduce the reported procedure and analysis. All materials are available at https://Github.com/saulgreenberg/Timelapse and http://saul.cpsc.ucalgary.ca/timelapse/.

## Data Availability

Timelapse is an open source project written in C#/WPF and available on the Github repository: https://github.com/saulgreenberg/Timelapse. For even easier access, Timelapse software, installation instructions, tutorial documentation (describing all its functions and including example image and template files), and mailing list information are freely available at http://saul.cpsc.ucalgary.ca/timelapse. Project managers and analysts are invited to download Timelapse, and developers are invited to modify or enhance the software as needed. Finally, Timelapse is actively maintained and supported by the first author of this paper. Contact saul@ucalgary.ca for more information.

## ENDNOTES

1 Timelapse availability is described at the end of this paper.

2 The cited paper concerns an earlier version of Timelapse. While it includes descriptions of a subset of interface features explored in this paper, those descriptions tend to be more superficial, are not provided as design patterns, or discussed in that context. The current paper also covers design patterns of features not present in that earlier system or paper.

3 A narrated video illustrating many of the Timelapse features described in this paper can be found at: http://grouplab.cpsc.ucalgary.ca/grouplab/uploads/Publications/Publications/2019-DesigningCameraTrapSoftware.TechSymp.mp4

4 An *image set* as the *collection of images* being analyzed by an analyst. The images that comprise an image set depends on how the project manager conceptualizes and manages images as a set. As one example, an image set can comprise only the images just extracted from the SD card at a particular camera station at the end of a servicing period. Alternately, an image set can comprise all images at a particular station, where new images extracted from a servicing period are added to the collection of older (perhaps already analyzed) images. As a further example, an image set can comprise all images taken from multiple camera stations at a particular study site.

5 This paper uses a deliberately simple schema/template for illustration. In practice, schemas can be quite rich and complex.
